# Tumor Microenvironment‐Responsive Nanoparticles Enhance IDO1 Blockade Immunotherapy by Remodeling Metabolic Immunosuppression

**DOI:** 10.1002/advs.202405845

**Published:** 2024-12-11

**Authors:** Mengna Wang, Yuhong Liu, Yanshi Li, Tao Lu, Min Wang, Zhaobo Cheng, Lin Chen, Tongling Wen, Min Pan, Guohua Hu

**Affiliations:** ^1^ Department of Otorhinolaryngology The First Affiliated Hospital of Chongqing Medical University Chongqing 400016 P. R. China; ^2^ The First Clinical College Chongqing Medical University Chongqing 400016 P. R. China

**Keywords:** chemodymic therapy, immunosuppression, indoleamine 2,3‐dioxygenase 1, KYN/TRP metabolism

## Abstract

The clinical efficacy of immune checkpoint blockade (ICB) therapy is significantly compromised in the metabolically disordered tumor microenvironment (TME), posing a formidable challenge that cannot be ignored in current antitumor strategies. In this study, TME‐responsive nanoparticles (HMP1G NPs) loaded with 1‐methyltryptophan (1‐MT; an indoleamine 2,3‐dioxygenase 1 [IDO1] inhibitor,) and S‐nitrosoglutathione (GSNO; a nitric oxide donor) is developed to enhance the therapeutic efficacy of 1‐MT‐mediated ICB. The HMP1G NPs responded to H^+^ and glutathione in the TME, releasing Mn^2+^, GSNO, and 1‐MT. The released Mn^2+^ catalyzed the production of abundant reactive oxygen species and nitric oxide from hydrogen peroxide and GSNO, and the generated nitric oxide, synergistically with 1‐MT, inhibited the accumulation of kynurenine mediated by IDO1 in the tumor. Mechanistically, HMP1G NPs downregulated tumor cell‐derived IDO1 via the aryl hydrocarbon receptor/signal transducer and activator of transcription 3/interleukin signaling axis to improve kynurenine/tryptophan metabolism and immunosuppression. In a murine breast cancer model, treatment with HMP1G NPs elicited effective antitumor immunity and enhanced survival outcomes. This study highlights a novel nano‐platform that simultaneously improves metabolism and enhances ICB efficacy to achieve a new and efficient antitumor strategy.

## Graphical abstract

The intelligent HMP1G NPs respond to H^+^ and glutathione in the tumor microenvironment (TME), They downregulate tumor‐derived IDO1 via the AhR/STAT3/IL axis, improve the KYN/TRP metabolic imbalance, and effectively counter immunosuppression in the TME. This work emphasizes a novel nanoplatform that combines CDT, nitric oxide, and metabolic improvement for an efficient clinical anti‐tumor immunotherapy.

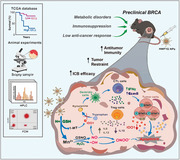

## 1 Introduction

Immune checkpoint blockade (ICB) therapy has been increasingly applied to a variety of solid tumors, significantly improving clinical outcomes in certain malignancies.^[^ ^1^, ^2^ ^]^ However, ICB therapy lacks efficacy for so‐called “cold” tumors, such as breast cancer (BRCA) and head and neck squamous carcinoma (HNSCC), as the intrinsic changes in tumor cells, including enzyme activity and metabolic disorders, can prevent immune cell infiltration or function within the tumor microenvironment (TME), leading to primary resistance to ICB therapy.^[^ ^3^, ^4^, ^5^ ^]^ Immunosuppression in solid tumors is closely associated with the altered metabolism of tumor cells.^[^ ^6^ ^]^ Therefore, strategies aimed at disrupting these metabolic pathways and reprogramming the immunosuppressive TME are crucial for expanding the applications of cancer immunotherapy.

Consumption of the essential amino acid tryptophan (TRP) and the accumulation of kynurenine (KYN) directly affect the function of effector T cells (Teffs), strongly promoting peripheral immune tolerance.^[^ ^7^, ^8^, ^9^ ^]^ Indoleamine 2,3‐dioxygenase 1 (IDO1)‐mediated TRP metabolism in the KYN pathway is among the most widely studied metabolic pathways. IDO1, as an immune checkpoint protein, is a necessary enzyme that catalyzes TRP degradation and KYN accumulation.^[^ ^8^, ^10^ ^]^ The upregulation of IDO1 expression has been observed in various solid tumors, making it an attractive immunotherapeutic target. The immunosuppression induced by KYN is attributed to the interaction of KYN and its derivatives with the aryl hydrocarbon receptor (AhR), which activates AhR signaling.^[^ ^11^, ^12^ ^]^ Activation of AhR signaling has multifaceted effects on immune regulation, including upregulation of IDO1 expression in tumor cells, upregulation of forkhead box P3 (FOXP3) expression in CD4^+^ T cells, attenuation of CD8^+^ T cell proliferation, and a reduction in the expression levels of interferons and granzymes released by these cells, ultimately increasing the ratio of immunosuppressive infiltration.^[^ ^12^, ^13^, ^14^ ^]^ IDO‐specific competitive protein inhibitors, such as 1‐methyltryptophan (1‐MT), can effectively inhibit IDO1 activity and alleviate immunosuppression.^[^ ^15^ ^]^ However, because of the low immunogenicity of solid tumors, single‐agent IDO blockade therapy has limited efficacy.^[^ ^16^ ^]^

It has been documented that immunogenic cell death can promote antigen presentation by releasing damage‐associated molecular patterns, thereby activating tumor‐specific immune responses and reversing the low immunogenicity of “cold” tumors.^[^ ^5^ ^]^ This strategy of enhancing immunogenicity is primarily achieved through the induction of cellular organelles or cellular oxidative stress, as observed in therapies, such as radiotherapy, photodynamic therapy, and sonodynamic therapy. However, these strategies inevitably cause severe damage to surrounding tissues.^[^ ^17^, ^18^ ^]^ Chemodynamic therapy (CDT), which involves a Fenton‐like reaction, converts hydrogen peroxide (H_2_O_2_) into highly toxic hydroxyl radicals (·OH) and is recognized as an emerging and effective reactive oxygen species (ROS)‐dependent cancer treatment strategy that effectively enhances immunogenicity and activates tumor‐specific immune responses.^[^ ^19^, ^20^, ^21^ ^]^ Additionally, researches have indicated that nitric oxide (NO) plays a crucial role in the formation and progression of cancer.^[^ ^22^ ^]^ NO can generate reactive nitrogen species as a multifunctional signaling molecule with direct effects on biomolecules. More importantly, NO can react with ROS to generate peroxynitrite (ONOO^−^), thus directly killing malignant tumor cells,^[^ ^23^, ^24^ ^]^ but also reversibly inhibiting the activity of IDO1, regulating the tumor immune microenvironment.^[^ ^24^ ^]^ Therefore, it is reasonable to consider that the combination strategy of CDT with NO treatment may improve the results of ICB antitumor immune therapy related to IDO1 blockade.

However, IDO1 inhibitors also face challenges, such as poor water solubility, low bioavailability, and lack of tumor specificity.^[^ ^16^ ^]^ Additionally, combination therapies often require different drugs to be effectively delivered to the tumor region in a spatiotemporally controlled manner. With the rapid development of nanotechnology, smart nano drug delivery systems can effectively address these issues.^[^ ^25^, ^26^ ^]^ By designing TME‐responsive smart nano drug delivery systems, drug release within tumors can be effectively controlled in the TME.^[^ ^27^ ^]^ Recently, hollow mesoporous manganese dioxide has received widespread attention. As a TME‐responsive nanodrug, MnO_2_ degrades in the acidic TME and reacts with glutathione (GSH) to generate the Fenton‐like agent, Mn^2+^, which catalyzes the generation of ·OH from H_2_O_2_ in the tumor, thereby driving the process of chemodynamic therapy.^[^ ^28^, ^29^ ^]^ These characteristics make MnO_2_ a highly suitable candidate vector for combined immunotherapy with CDT, NO, and IDO1 inhibitors.

**Figure 1:**
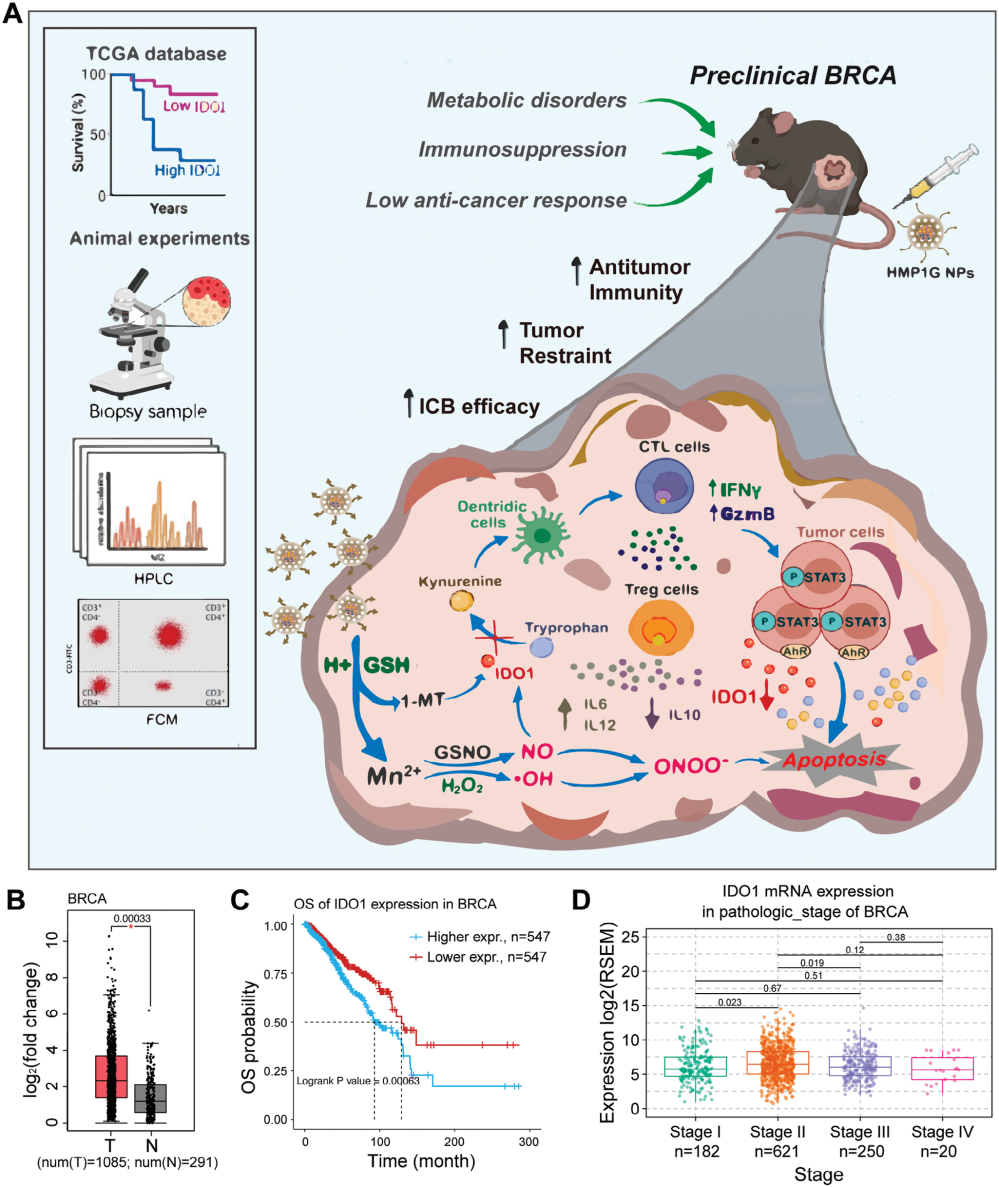
Bioinformatics analysis of IDO1 in BRCA and the effect of nano‐systems in reversing metabolic disorders and immunosuppression in the TME of BRCA. A) Schematic representation of HMP1G NPs‐mediated CDT and nitic oxide‐treatment combination strategy in a BRCA mouse model of the TME. NPs downregulate IDO1 protein levels in the TME via the AhR/STAT3/IL axis to improve KYN/TRP metabolism and immunosuppression. B) Box plot depicting the expression of IDO1 in BRCA patients (n = 1085) and healthy individuals (n = 291) analyzed from the TCGA database. C) Survival curves of BRCA patients with high (n = 547) and low (n = 547) IDO1 expression levels. D) Box plot illustrating the correlation between IDO1 mRNA levels and BRCA pathological staging.

Here, we designed a TME‐responsive nanodrug HMnO_2_‐PEG@1‐MT@GSNO nanoparticles (named HMP1G NPs) by loading the IDO1 inhibitor 1‐MT and the NO donor GSNO onto polyethylene glycol‐modified HMnO_2_ (HMP). This approach aims to enhance tumor immunogenicity in response to an acidic TME, inhibit IDO1 expression to ameliorate immune tolerance and reprogram the immunosuppressive metabolic environment to enhance antitumor efficacy. In the poorly immunogenic BRCA animal models (**Figure** **1**A), HMP1G NPs were disassembled in specific acidic TME to achieve pH‐triggered on‐demand drug release within the tumor. This led to extensive oxidative stress in TME, blockade of KYN metabolism accumulation, and inhibition of both 4T1 orthotopic tumor growth and artificial metastasis progression in mice. The nanodrug constructed in this study functioned through the following steps: First, the smart nanodrug responded to H^+^ and GSH in the TME, generating Mn^2+^ and releasing the NO donor GSNO and 1‐MT to promote Fenton reaction‐associated CDT. Second, the generated Mn^2+^ catalyzed endogenous H_2_O_2_ production to generate abundant ·OH, while triggering GSNO decomposition to release NO. Third, the released NO not only synergistically induces tumor oxidative stress with ·OH but also collaborates with released 1‐MT to inhibit IDO1 expression. This helped improve the metabolic immunosuppressive TME to reprogram it into an immunogenic phenotype, enhancing dendritic cell (DC) antigen presentation, activating cytotoxic T lymphocyte (CTL) function, inhibiting negative regulation by regulatory T (Treg) cells, and increasing the 1‐MT‐mediated ICB response rate. Mechanistically, our research revealed that, in “cold” solid tumors, the excessive expression of IDO1 derived from tumor cells leads to the accumulation of KYN, which activates interleukin (IL) family signaling through the aryl hydrocarbon receptor/signal transducer and activator of transcription 3 (AhR/STAT3), exerting systemic effects on the malignant progression of tumors and antitumor immune resistance. The successful construction of multifunctional HMP1G NPs significantly decreased IDO1 protein levels. Importantly, HMP1G did not have an off‐target effect activated of AhR, which is driven by traditional IDO1 inhibitors. In summary, HMP1G nanoparticles downregulated tumor‐derived IDO1 via the AhR/STAT3/IL axis, ameliorating KYN/TRP metabolic dysregulation, effectively overcoming the metabolic immune suppression in the TME, reversing the immune tolerance of cold tumors, and making tumor cells sensitive to 1‐MT‐mediated ICB. We emphasize the potential of intelligent nanosystems to modulate the immunosuppressive TME. Our experimental results indicate that this specific, effective, and highly biocompatible nanoplatform has the potential for clinical applications in cancer therapy.

## 2 Results

### 2.1 The Synthesis and Characterization of IDO1‐Inhibiting Nano Drug HMP1G NPs

**Figure 2:**
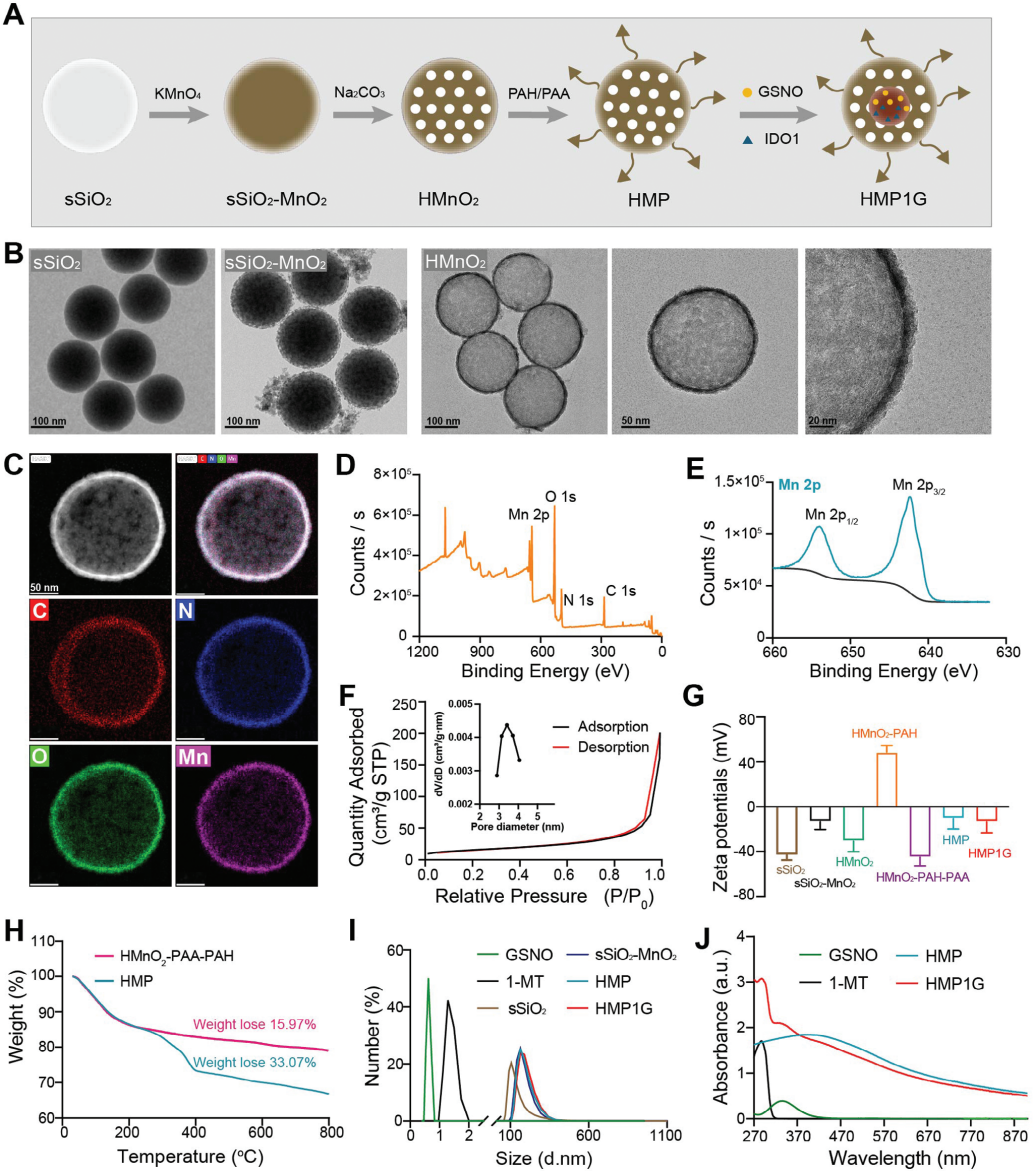
Schematic diagram and characterization of HMP1G NPs. A) Illustration of the stepwise synthesis of HMP1G NPs and subsequent drug loading process. B) Representative TEM image of sSiO_2,_ sSiO_2_‐MnO_2,_ and HMnO_2_ NPs. (Scale bar: 100, 50, 20 nm) C) Representative HAADF‐STEM image of HMnO_2_ NPs and corresponding elemental mapping. (Scale bar: 50 nm) D) XPS survey spectrum and E) high‐resolution scans of Mn 2p peaks in HMnO_2_. F) Pore‐size distribution curve and N2 adsorption/desorption isotherms (inset) of HMnO_2_. G) Changes in Zeta potential for SiO_2_, sSiO_2_‐MnO_2_, HMnO_2_, HMnO_2_‐PAH, HMnO2‐PAH/PAA, HMP, and HMP1G. H) Thermogravimetric curves of HMnO_2_‐PAA‐PAH and HMP. I) Particle size distribution of GSNO, 1‐MT, SiO_2_, sSiO_2_‐MnO_2_, HMP, and HMP1G. J) UV–vis–NIR absorption spectra of GSNO, 1‐MT, SiO_2_, sSiO_2_‐MnO_2_, HMP, and HMP1G.

In recent years, ICB therapy has shone brightly in the landscape of precision cancer treatment strategies, particularly for breast cancer.^[^ ^5^ ^]^ The activating of CD8^+^ T cell recruitment can provide unprecedented opportunities for the development of immunotherapies for BRCA.^[^ ^3^ ^]^ IDO1, an endogenous immune inhibitory mediator, stimulates the accumulation of Treg cells and inhibits T cell activity by consuming TRP and accumulating KYN in the TME. TCGA bioinformatics analysis revealed that, compared to normal breast tissue (291 cases), IDO1 expression is significantly elevated in the breast tumors of BRCA patients (1085 cases) (Figure 1B). Importantly, survival analysis based on differential levels of IDO1 expression revealed that IDO1^low^ BRCA patients exhibited a greater survival advantage (Figure 1C), and high IDO1 expression correlated with the clinical staging of BRCA (Figure 1D). However, previous preclinical trials have had relatively few studies focused on improving IDO1 expression. To address the above problems, we designed a nano drug based on HMP1G NPs for TME‐responsive drug delivery to ameliorate the dysregulation of KYN/TRP metabolism and immunosuppression within BRCA. The synthesis of HMP1G nanoparticles (NPs) is illustrated in **Figure** **2**A. In summary, monodispersed silica nanoparticles were synthesized through the hydrolysis of tetraethyl orthosilicate (TEOS) and were immediately employed as hard templates. A uniform sSiO_2_‐MnO_2_ structure was achieved by mixing the freshly prepared silica nanoparticles with potassium permanganate (KMnO₄) for several hours, followed by etching in a Na₂CO₃ solution to yield hollow manganese dioxide nanoparticles (HMnO₂). To enhance the water solubility and physiological stability of the HMnO₂ nanoshells, modified with polyethylene glycol (PEG). In this process, the negatively charged HMnO₂ nanoshells were encapsulated by the cationic polymer poly(allylamine hydrochloride) (PAH), followed by the application of the anionic polymer poly(acrylic acid) (PAA) through electrostatic interactions. Amino‐terminated PEG (NH₂‐PEG) was conjugated to the PAA‐coated HMnO₂ nanoshells via amide bonding, resulting in the formation of HMnO₂‐PEG nanoshells. Finally, GSNO and 1‐MT were co‐loaded into the hollow structure of HMnO_2_‐PEG nanoshells to obtain HMP1G NPs for further experiments.

**Figure 3:**
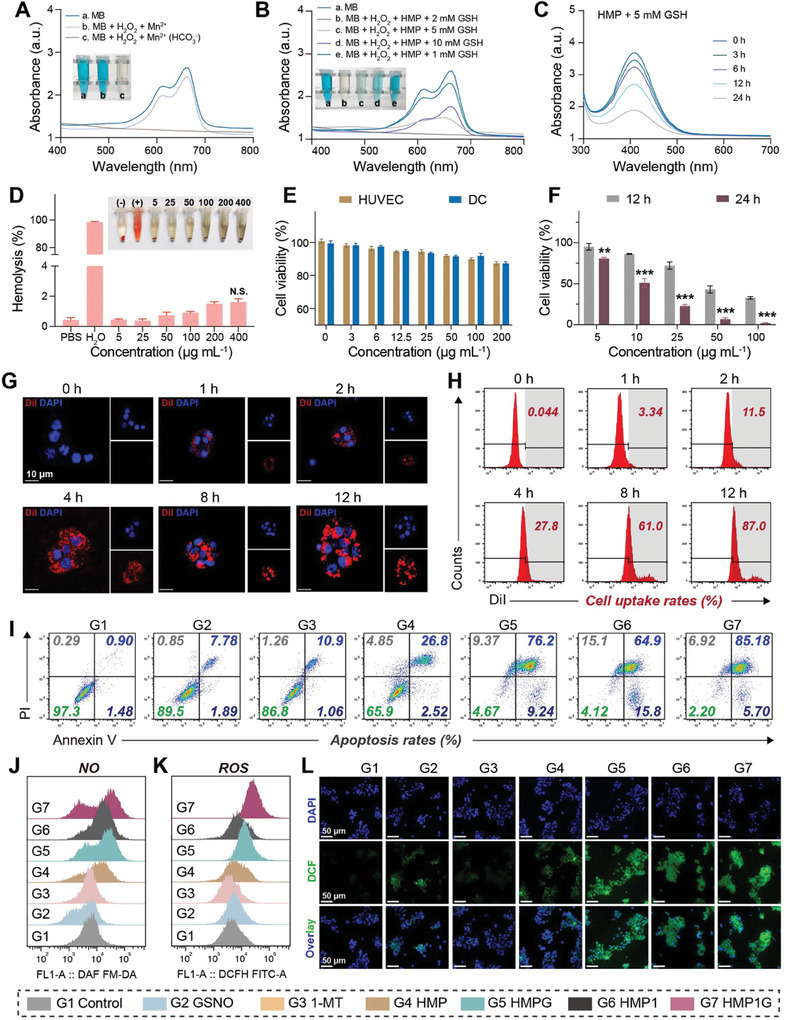
NPs‐mediated CDT effect and in cell experiments in vitro. A) Representative UV–vis spectrums and digital photos(inset) of MB degraded by Mn^2+^ mediated Fenton‐like reactions in different solutions. B) Representative UV–vis spectrums and digital photos(inset) of NPs treated with H_2_O_2_ and GSH for MB degradation (inset). C) Representative UV–vis spectrums of HMP incubates with GSH at different time points. D) Concentration‐dependent hemolysis and relative digital photo (inset) of HMG1P NPs. H_2_O and PBS are set as positive and negative controls, respectively. E) Relative cell viability of HUVEC and DC cells treated with different concentrations of HMP1G NPs for 24 h. F) Relative cell viability of 4T1 cells treated with different concentrations of HMP1G NPs for 12 and 24 h. G) CLSM images of 4T1 cells incubated with HMP NPs attached to DiI at different times and H) corresponding FCM images (Scale bar: 10 µm). I) Apoptosis FCM images of 4T1 cells treated with different groups for 24 h. J) NO FCM images of 4T1 cells treated with different groups for 24 h. K) ROS FCM images and L) corresponding CLSM images of 4T1 cells treated with different groups for 24 h (Scale: 50 µm). All data are presented as mean ± standard deviation (n = 6), where ^*^ is the comparison between this group and the control group, # is the comparison between this group and the 1‐MT group, N.S. represents the comparison between this group and the control group is meaningless, ^*^ *p* ≤ 0.05, ^**^ *p* ≤ 0.01, ^**^ *p* ≤ 0.001.

Transmission electron microscopy (TEM) images reveal the spherical morphology of sSiO_2_, sSiO_2_‐MnO_2,_ and a hollow structure of HMnO_2_ (Figure 2B). Elemental mapping analysis further confirmed the presence and uniform distribution of C, N, O, and Mn elements within the nanostructures (Figure 2C). As shown in Figure S1 (Supporting Information), the natural structure of the sSiO_2_ was investigated through X‐ray diffraction analysis. The patterns of the SiO_2_ corresponded to the standard data for SiO_2_ (PDF#97‐001‐6336). Additionally, the elemental composition of the sSiO_2_ and sSiO_2_‐MnO_2_ were further characterized using X‐ray photoelectron spectroscopy (XPS). The XPS results confirmed the existence of Si and O elements in SiO_2_, while sSiO_2_‐MnO_2_ exhibited the coexistence of Si, O, and Mn elements (Figures S2 and S3, Supporting Information). Furthermore, XPS was used to verify the elemental composition of HMnO_2_ and analyze the chemical valence state of Mn, the binding energy centers at 653.83 and 642.29 eV were indexed to the high‐resolution XPS peaks of Mn 2p_1/2_ and Mn 2p_3/2_, respectively (Figure 2D,E). Subsequently, the specific surface area and pore volume of HMnO_2_ were analyzed through N_2_ adsorption‐desorption isotherms and pore size distribution, revealing a surface area and average pore diameter of 55.8 m^2^ g^−1^ and 3.6 nm, respectively, making it a promising nano‐carrier for efficient drug loading (Figure 2F) The successful fabrication of HMP1G NPs was demonstrated through the observed changes in ζ‐potential during the stepwise synthesis process (Figure 2G). Thermal gravimetric analysis (TGA) of HMnO_2_‐PAA‐PAH and HMP NPs confirmed the successful modification with NH_2_‐PEG5000 at an approximate modification rate of 17.03% (**Figure** **3**H). Furthermore, successful loading of 1‐MT and GSNO was confirmed by UV–vis absorption spectroscopy, with encapsulation efficiencies of ≈88.7 and 84.5%, respectively (Figure 2J). The release behavior of 1‐MT and GSNO from HMP1G NPs was studied in solutions with different conditions (Figure S4, Supporting Information). Compared with the slow drug release curve of HMP1G NPs at pH 7.4, the release rates of 1‐MT and NO were faster in weakly acidic solutions (pH 6.5), especially containing GSH (5 mm), because of the acidic and GSH‐triggered decomposition of HMnO_2_ into Mn^2+^ ions. Additionally, dynamic light scattering measurements after loading of GSNO and 1‐MT revealed average particle sizes of ≈187.7 and 193.6 nm for HMP NPs and HMP1G NPs, respectively (Figure 2I). The colloidal stability of nanomaterials is a critical parameter for assessing their suitability for intravenous (*i.v*.) administration. Notably, HMP1G NPs exhibited excellent stability in culture media over a period of 14 days (Figure S5, Supporting Information). These physical properties of the HMP1G NPs, including their nanoscale size and high dispersibility, are advantageous for conducting in vitro performance assessments and biomedical applications in vivo.

### 2.2 HMP1G NPs Exhibited Excellent CDT‐Mediated Antitumor Effects In Vitro

HMP NPs remain stable under physiological pH conditions but may degrade into Mn^2+^ ions under reduced pH.^[^ ^20^, ^29^ ^]^ Therefore, we first evaluated the degradation behavior of HMP1G NPs in different environments (Figure S6, Supporting Information). As expected, even after 24 h, HMP NPs exhibited no significant changes in the physiological environment (pH 7.4), but displayed degradation behavior in a time‐dependent manner under acidic conditions (pH 6.5, pH 6.5 + GSH [5mm]). Subsequently, the NO‐responsive release from HMP1G NPs was detected using the Griess reaction. As shown in Figure S7 (Supporting Information), no significant NO release was observed in the physiological environment. In contrast, rapid NO release was observed from HMP1G NPs in an acid environment (pH 6.5 + GSH [5mm]), reaching a plateau after 8 h, which is attributed to the degradation of HMP1G NPs and the release of Mn^2+^ and GSNO in the TME, with GSNO further decomposing and releasing NO under the driving force of Mn^2+^, indicating that HMP1G NPs exhibit excellent stability during blood circulation, reduce drug leakage, and ensuring effective delivery of NO prodrugs to tumor tissues.

In the presence of Fenton‐like Mn^2+^, HCO_3_^−^ exhibits ·OH production activity, while GSH has a scavenging effect on ·OH. We chose methylene blue (MB) as an indicator of ·OH formation and investigated the generation of ·OH by Mn^2+^ in the presence of HCO_3_^−^ through a Fenton‐like reaction and depletion of GSH.^[^ ^30^, ^31^ ^]^ First, we reacted MnCl_2_, MB, and H_2_O_2_ in a buffer solution containing HCO_3_^−^ for 30 min and found a significant decrease in the absorbance of MB. Conversely, no significant changes in MB absorbance were observed in the buffer solution without HCO_3_^−^. The rapid degradation of MB by Mn^2+^ and H_2_O_2_ with the assistance of HCO_3_^−^ indicated that the Mn^2+^‐driven Fenton‐like reaction effectively generated ·OH (Figure 3A). GSH can promote the degradation of HMP, driving the Mn^2+^‐mediated Fenton‐like reaction, while GSH can eliminate the ·OH formed, thereby limiting the efficiency of CDT. We investigated the degradative capacity of HMP NPs for MB at GSH concentrations ranging from 1 to 10 mm (representative of GSH concentrations in tumors). As shown in Figure 3B, when the GSH concentration was low (1 mm), the degradation of MB was insignificant, which was attributed to the limited degradation rate of HMP at low GSH concentrations, leading to an insufficient release of Mn^2+^. Furthermore, the degradation rate peaked at 2 mm and subsequently declined as the GSH concentration increased from 2 to 10 mm, because an excess of GSH could scavenge the generated ·OH, converting it to GSSG, and reducing the degradation of MB. Meanwhile, We collected and freeze‐dried the buffer solution containing HMP NPs and GSH (5mm) for further validation. According to the XPS (Figure S8, Supporting Information), two strong binding energy peaks at 641.4 eV (Mn^2+^ 2p3) and 653.11 eV (Mn^2+^ 2p1) corresponded to Mn^2+^, which directly confirms the production of Mn^2+^ ions by HMP NPs in an acidic environment. Upon co‐incubation of HMP NPs with GSH, we observed a gradual decrease in the GSH content in the system over time (Figure 3C). This indicated that HMP NPs can disrupt the antioxidant imbalance in tumors and enhance the efficacy of CDT by consuming GSH.

The above class of Fenton reactions are described in detail by the following equations:

(1) $$\mathrm{Mn}O_{2}+\mathrm{GSH}\to Mn^{2+}+\mathrm{GSSG}$$

(2) $$Mn^{2+}+H_{2}O_{2}+{\mathrm{HCO}}_{3}^{-}\to •\mathrm{OH}$$

Biosafety is the foundation for nanodrugs conducting biological experiments. Even at a concentration of 400 µg mL^−1^, the rate of hemolysis of red blood cells induced by HMP1G NPs was less than 3%, ensuring the safety of HMP1G NPs for clinical use (Figure 3D). Furthermore, hematological and serum biochemical analyses of mice were administered with HMP1G NPs via *i.v*. Injection on days 1, 7, 14, and 21 showed no significant changes compared to those of normal mice. Histological staining of major organs also revealed no pathological abnormalities, indicating the excellent tissue compatibility of the HMP1G NPs (Figures S9 and S10, Supporting Information).

By employing standard Cell Counting Kit‐8 (CCK8) analysis, we initially confirmed that regular HMP1G NPs exhibited negligible cytotoxicity toward normal cells (such as DCs) and human umbilical vein endothelial cells (HUVECs) (Figure 3E), demonstrating the outstanding biocompatibility of HMP1G NPs for safe *i.v*. administration. Subsequently, we evaluated the cytotoxicity of HMP1G NPs in 4T1 cancer cells. As shown in Figure 3F, after co‐incubation with 50 µg mL^−1^ of HMP1G NPs and 4T1 cells for 24 h, cell viability decreased to below 10%. When the drug concentration of HMP1G NPs was increased to 100 µg mL^−1^, the cytotoxicity reached more than 98%. We used an HMP1G NPs concentration of 50 µg mL^−1^ for subsequent cell experiments. Evaluation of the tumor cell phagocytic characteristics of the nano‐system using DiI‐labeled HMP NPs revealed widespread red fluorescence distributed within 4T1 cells after 4 h of co‐incubation, indicating good intracellular uptake (Figure 3G). This trend further increased after 12 h of incubation, as indicated by stronger red fluorescence. Qualitative results from flow cytometry (FCM) (Figure 3H; Figure S11, Supporting Information) demonstrated a similar cellular uptake trend, with the cellular uptake rate of HMP NPs reaching as high as 87% after 12 h of incubation. The outstanding cellular internalization efficiency of the HMP NPs contributed to the eradication of tumor cells.

Subsequently, we used annexin V‐FITC/propidium iodide (PI) staining to precisely monitor the apoptosis ratio of 4T1 cells co‐cultured with various NPs using FCM. As shown in Figure 3I and Figure S12 (Supporting Information), lower levels of apoptosis were observed in HMP NPs than in the control after 24 h of incubation (33.1%). Compared to the specific cell damage mediated by free 1‐MT (apoptotic value of 13.1%), HMP1 exhibited significantly enhanced cytotoxicity induced by its excellent drug delivery efficiency. The apoptotic efficiency of GSNO or HMP NPs alone was unsatisfactory. When loaded with GSNO, HMP1G NPs induced severe cell damage, reaching the highest apoptotic efficiency of 96.82% after 24 h. We employed a standard GSH assay kit to evaluate the GSH depleting effect of nano‐system, as shown in Figure S13 (Supporting Information), HMP1G NPs effectively depleted the 4T1 intracellular GSH content, significantly outperforming other groups. Next, we observed significant NO and ROS generation in the HMP1G NPs group by FCM (Figure 3J,K). Figure 3L demonstrates abundant ROS fluorescence within the HMP1G NPs group. These qualitative and quantitative results indicate that the generation of NO and ROS by HMP1G NPs is the reason for their outstanding anticancer cytotoxicity. Taken together, these results confirmed that HMP1G NPs possess an excellent CDT effect and NO therapy, achieving high‐efficiency tumor‐killing capabilities, and thereby providing an experimental basis for subsequent in vivo models.

### 2.3 HMP1G NPs Downregulate IDO1 Enzyme and Activate Immune Effects via the AhR/STAT3/IL Axis

**Figure 4:**
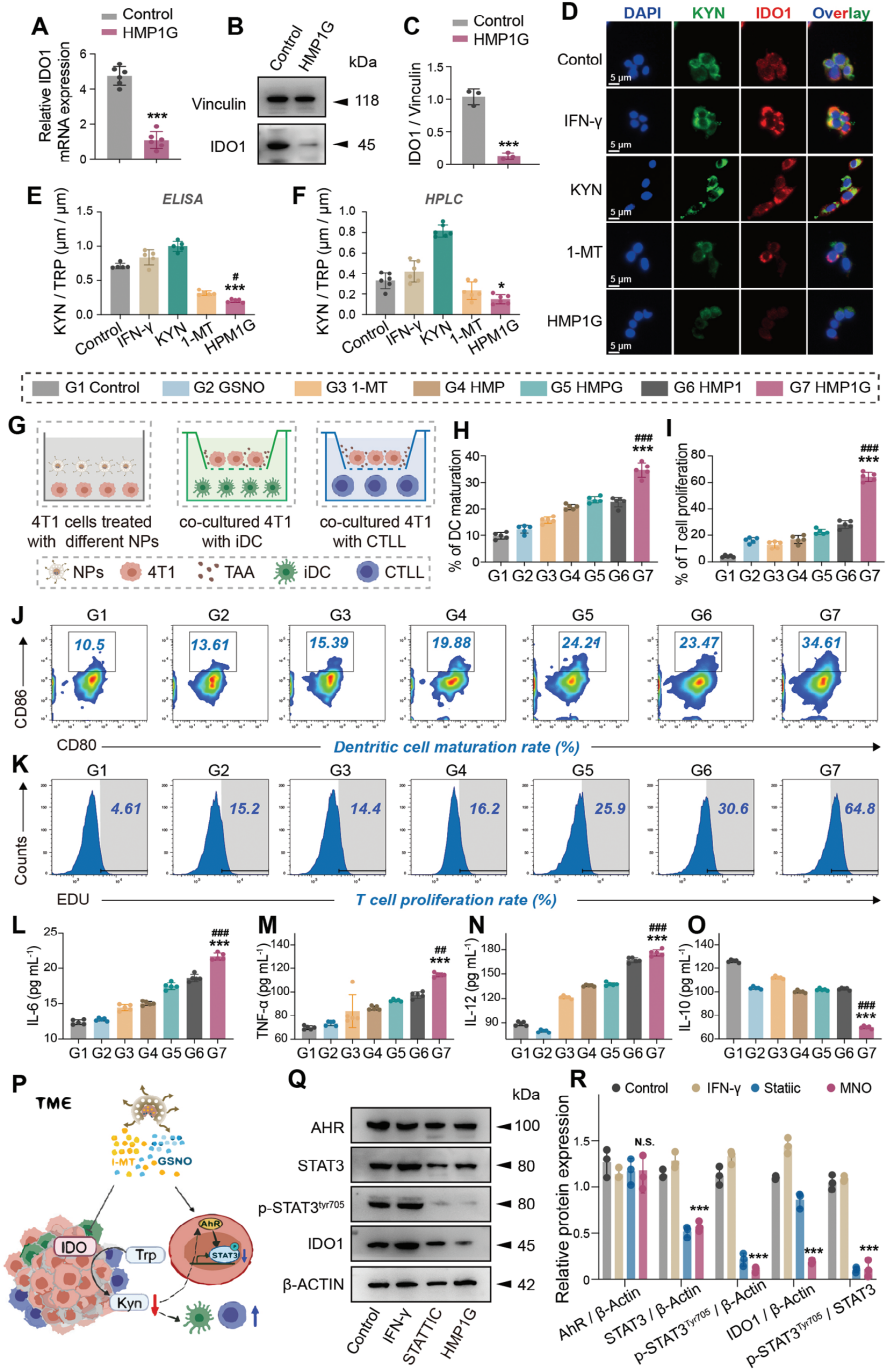
NPs downregulate IDO1 through the AHR/STAT3/IL axis to improve KYN/TRP metabolism and immune suppression. Qualitative and quantitative analysis of IDO1 mRNA and protein levels in 4T1 cells treated with HMP1G using qRT PCR A) and WB B) C). D) Immunofluorescence of 4T1 cells treated with different groups, Alexa Fluor 488 was used to label KYN, Alexa Fluor 555 for KYN, and DAPI for nucleus (Scale bar: 5 µm). Determine the KYN and TRP levels of 4T1 cells supernatants in different treatment groups using E) Elisa and F) HPLC‐MS/MS, and calculate the corresponding KYN/TRP ratio (n = 6 biological replicates). G) Co‐culture pattern of 4T1 cells with immune cells, 4T1 cells were pre‐incubated with different NPs for 24 h. Maturation FCM J) and quantitative analysis H) of DC cells co‐cultured with processed 4T1 cells. Flow cytometry K) and quantitative analysis I) of the proportion of EdU^+^ T cells co‐cultured with processed 4T1 cells. Collect the supernatant of DC cell culture medium from each treatment group and use ELISA to identify inflammatory factors such as L) IL‐6, M) TNF‐α, N) IL‐12, and O) IL‐10. P) Schematic diagram of NPs regulating IDO1 in TME to improve metabolism and immunosuppression. Q) Evaluate AHR, STAT3, and p‐STAT3^Tyr705^ protein levels by immunoblotting and R) corresponding quantitative analysis. All data are presented as mean ± standard deviation (n = 6), where ^*^ is the comparison between this group and the control group, # is the comparison between this group and the 1‐MT group, N.S. represents the comparison between this group and the control group is meaningless, ^*^ *p* ≤ 0.05, ^**^ *p* ≤ 0.01, ^**^ *p* ≤ 0.001; # *P* ≤ 0.05, ## *p* ≤ 0.01, ### *p* ≤ 0.001.

To investigate the potential of HMP1G NPs as novel ICB formulations for BRCA clinical applications, we assessed the inhibitory effect of nano‐systems on IDO1 enzyme by western blot in vitro. It is clear that compared to the normal mouse mammary epithelial cell line HC11, a significant upregulation of IDO1 expression in the breast cancer cell lines 4T1 (Figure S14, Supporting Information). Concurrently, there was almost no difference in IDO1 levels between the 4T1 cells treated with HMP NPs and the control group (PBS) (Figure S15, Supporting Information). This suggests the influence of HMP NPs on IDO1 expression was negligible. Compared with the control group (PBS), the HMP1G NPs group showed significant suppression of IDO1 expression and a marked decrease in mRNA levels (**Figure** **4**A–C). We then studied the IDO1 inhibitory activity of the HMP1G NPs on IDO1^high^ 4T1 cells, which were induced by incubating normal 4T1 cells with interferon‐gamma (IFN‐γ).^[^ ^32^ ^]^ Exogenous KYN was used as the quantitative standard. Cellular immunofluorescence (IF) revealed that after IFN‐γ stimulation, IDO1 and KYN levels increased, and the addition of exogenous KYN primarily upregulated KYN levels in 4T1 cells. Notably, compared with free 1‐MT inhibitors, HMP1G NPs demonstrated significantly more effective inhibition of IDO1 expression and the transformation of essential TRP to immunosuppressive KYN mediated by IDO1 (Figure 4D). These results suggested that HMP1G NPs can serve as effective and biocompatible IDO1 inhibitors. Furthermore, we used ELISA and high‐performance liquid chromatography‐tandem mass spectrometry (HPLC‐MS/MS) to analyze the culture supernatant to understand the metabolic status of the 4T1 cells. As shown in Figure 4E,F, HMP1G NPs effectively reduced the KYN/TRP ratio, thereby eliminating KYN levels.

DC uptake and processing of tumor‐associated antigens (TAAs) subsequently stimulates the activation of immature T cells and further induces their proliferation and differentiation.^[^ ^33^, ^34^ ^]^ To elucidate the activating effect of HMP1G NPs on immune cells, we constructed an in vitro co‐culture model consisting of 4T1 cells and immune cells (Figure 4G), to assess DC maturation and T cell proliferation. As depicted in Figure 4H,I, compared to the control group (PBS), treatment with 1‐MT, HMPG, or HMP1 resulted in a substantial increase in the proportion of mature CD86^+^ CD80^+^ DCs and EdU^+^ T cells, indicating improvements in antigen‐presenting DC activation and T cell proliferation. Notably, the HMP1G NPs group exhibited the most remarkable immune activation response, with a three‐fold increase in mature DC cells (34.61%) and a 4.5‐fold increase in proliferating T cells (64.8%) compared to the free 1‐MT group (Figure 4H–K). Furthermore, we used ELISA to examine the levels of inflammatory cytokines in the DC culture supernatants of each treatment group to explore. It was observed that following treatment with HMP1G NPs, the expression level of IL‐6, IL‐10, and TNF‐α was significantly higher than those of the other groups, and the secretion level of IL‐10 was also markedly reduced (Figure 4L–O). These antitumor inflammatory factors participate in immune responses by activating innate and adaptive immunity and binding to immunoreceptors to regulate T cell activation and proliferation. The exceptional immune activation demonstrated by HMP1G NPs can be attributed to several factors. First, the poor solubility of free 1‐MT limits its uptake efficiency and bioavailability. In contrast, HMP1G NPs possessed a higher drug‐loading capacity, effectively overcoming the poor solubility of 1‐MT and enhancing drug bioavailability. Second, 4T1 cells efficiently internalized HMP1G NPs in response to the additional ·OH generated by intracellular GSH and H_2_O_2_, resulting in the release of 1‐MT and NO. Therefore, the combined action of 1‐MT and NO can significantly inhibit IDO, activated DCs, and enhanced T cell proliferation. This confirmed the excellent potential of HMP1G NPs to enhance antitumor immune responses in vitro.

Dietary tryptophan can be metabolized into KYN by the activity of IDO1 in intestinal epithelial cells and DCs. Through the activation of AhR (a ligand‐activated transcription factor and immune sensor), a receptor closely associated with cancer progression, it promotes the differentiation of immature CD4^+^ T cells into Foxp3^+^ Tregs.^[^ ^10^, ^35^ ^]^ STAT3 is a key transcription factor that responds to pro‐tumor signals, effectively transcribing inflammatory cytokines associated with systemic metabolism.^[^ ^36^ ^]^ The phosphorylation of the Tyr705 site of STAT3 (p‐Tyr705) mediates the classical activation pathway of STAT3 and effectively suppresses the transcription of inflammatory cytokines associated with systemic metabolism, thereby promoting cancer cell proliferation and migration.^[^ ^37^, ^38^ ^]^ There is also evidence suggesting that STAT3, a positive regulator of AhR, is involved in regulating the abundance and function of Treg cells to weaken radiotherapy in HNSCC. In tumor cells, overactive STAT3 reduces the expression levels of immunostimulatory factors, including IFN‐γ and pro‐inflammatory cytokines (IL‐6, IL‐12, and TNF‐α), ultimately promoting the formation of an immunosuppressive TME.^[^ ^37^, ^39^ ^]^ Given that AhR is the canonical receptor for KYN, we hypothesized that these nano‐systems would improve the imbalance in KYN metabolism by inhibiting the AhR/STAT3/IL pathway.

Therefore, we proceeded to experimentally explore this hypothesis by inducing the overexpression of IDO1 and KYN in tumor cells with IFN‐γ stimulation and utilizing Stattic (a p‐STAT3 inhibitor that can inhibit STAT3 phosphorylation) as a positive control.^[^ ^40^ ^]^ As shown in Figure 4Q, IFN‐γ stimulation led to high levels of IDO1 and STAT3 protein phosphorylation in 4T1 cells. The addition of Stattic significantly inhibited the levels of STAT3 and its phosphorylation but did not improve the IDO1 phenotype. HMP1G NPs significantly downregulated the expression levels of IDO1, STAT3, and p‐STAT3^Tyr705^ without affecting AhR expression, supporting the downstream role of STAT3 in AhR. Notably, HMP1G NPs inhibited STAT3 expression, the efficacy equivalent to that of Stattic (Figure 4Q,R). This fully validated our hypothesis that in the absence of affecting AhR, HMP1G NPs downregulate STAT3 levels and phosphorylation at the Tyr705 site (Figure 4P), inhibiting the protein expression level of IDO1, thereby not only improving the imbalance in KYN/TRP metabolism, but also promoting the secretion of immunostimulatory factors, activating immune cell function and proliferation, and effectively improving immune responsiveness.

### 2.4 HMP1G NPs Exhibit Outstanding Antitumor Effects in the 4T1‐Tumor‐Bearing Mouse Model

**Figure 5:**
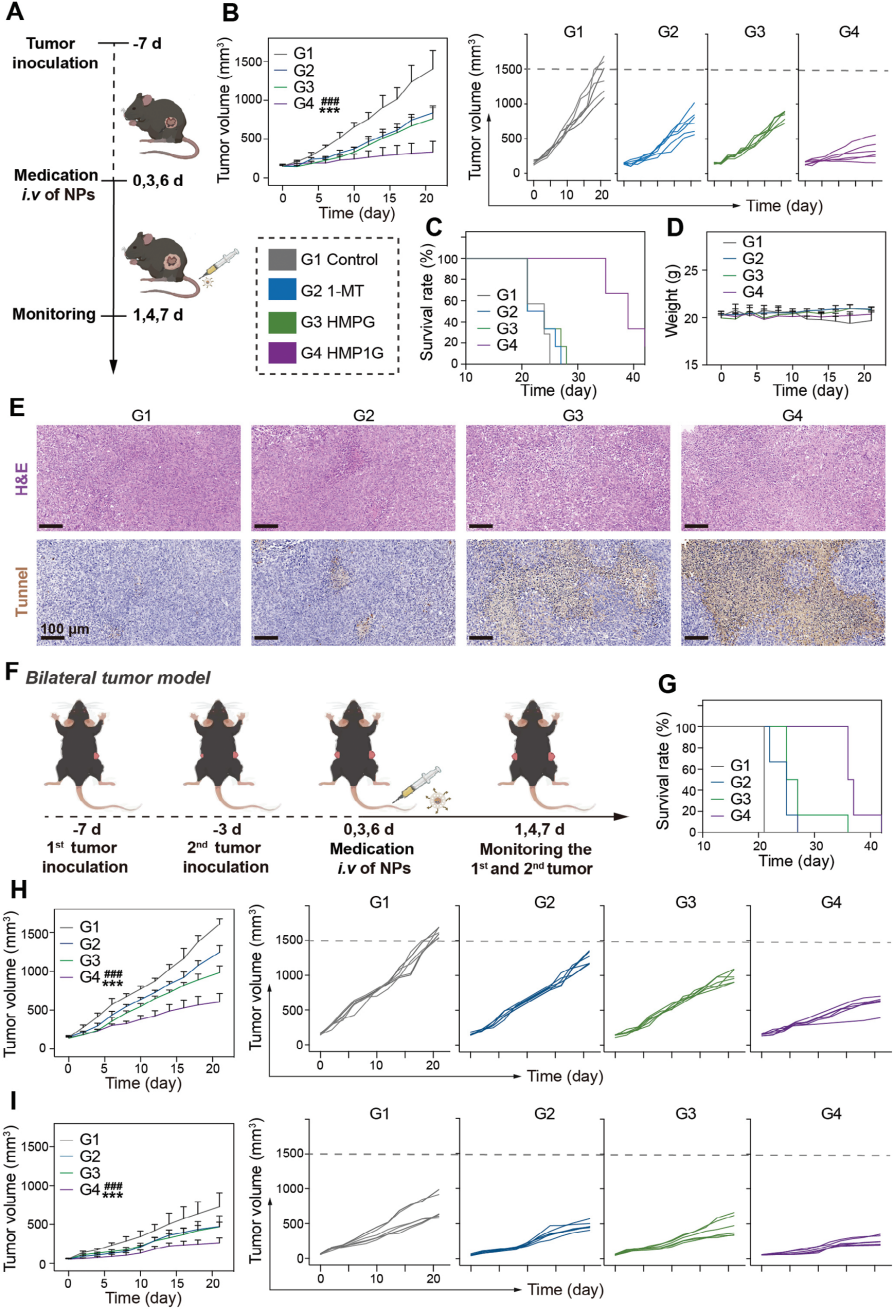
In vivo anti‐tumor therapeutic efficacy of NPs. A) Schematic representation of the experimental timeline for 4T1 tumor‐bearing mice. B) Mean tumor growth curves of mice (n = 6) subjected to various treatments along with individual tumor growth curves of each mouse. C) Survival curves of mice (n = 6) following different treatments. D) Mean body weights of 4T1 tumor‐bearing mice (n = 6) of different treatments. E) H&E and TUNEL staining of tumor sections collected post‐treatment completion (Scale bar: 100 µm). F) Experimental design for investigating the anti‐tumor abscopal effect of NPs using a bilateral 4T1 tumor model. G) Survival curves of 4T1 tumor‐bearing mice (n = 6) following various treatments. Mice were considered deceased when tumor volume reached 1500 mm^3^. Mean tumor growth curves and corresponding individual tumor growth curves of primary tumors H) and distant tumors I) in treatment groups. All data are presented as mean ± standard deviation (n = 6), where ^*^ is the comparison between this group and the control group, # is the comparison between this group and the 1‐MT group, N.S. represents the comparison between this group and the control group is meaningless, ^*^ *p* ≤ 0.05, ^**^ *p* ≤ 0.01, ^**^ *p* ≤ 0.001; # *P* ≤ 0.05, ## *p* ≤ 0.01, ### *p* ≤ 0.001.

Encouraged by the effective role of HMP1G NPs in reversing KYN/TRP metabolism and immunosuppressive effects, we meticulously assessed their anticancer efficacy in a low immunogenicity 4T1‐tumor‐bearing mouse model. Given the limitations of mono‐drug‐loaded nano‐systems for anticancer therapy, as demonstrated in in vitro experiments, we focused on the comparative analysis of free 1‐MT, HMPG, and HMP1G NPs at the in vivo level. The treatment regimens are shown in **Figure** **5**A. Initially, we randomly divided 24 tumor‐bearing C57 mice (≈150 mm^3^) into four groups (n = 6) administered the following treatments: 1) PBS; 2) 1‐MT; 3) HMPG NPs; and 4) HMP1G NPs. Mice in groups 2–4 received *i.v*. NPs medication treatments on days 0, 3, and 6 at equivalent doses of NPs (20 mg kg^−1^) and 1‐MT (6.6 mg kg^−1^). By monitoring tumor sizes, we observed that the inhibitory effect of the HMP1G NPs group on 4T1 tumor growth was significantly greater than that of the 1‐MT and HMPG groups (Figure 5B). Additionally, the median survival time for mice in the 1‐MT and HMPG groups was 25 days, with no significant improvement compared to that in the control group (Figure 5C), whereas two mice were still alive in the HMP1G NPs group at day 40. Furthermore, no significant weight changes were observed after any of the treatments (Figure 5D), indicating excellent biocompatibility of HMP1G NPs at the tested dose. Following the collection of the respective tumor tissues at the end of the treatment period, we conducted H&E and TdT‐mediated dUTP nick‐end Labeling (TUNEL) staining analyses to assess tumor apoptosis and necrosis. Compared with the untreated group, the HMP1G NPs group exhibited the most severe cell apoptosis and necrosis, while groups 2 and 3 showed moderate levels of cell apoptosis and necrosis (Figure 5E). Overall, these results suggested that HMP1G NPs demonstrated excellent anticancer effects, without causing significant side effects.

Inspired by the remarkable performance of HMP1G NPs in anticancer therapy, we evaluated the abscopal effects of HMP1G NPs on inhibiting distant tumor growth. We established an artificial metastasic tumor to represent distant tumors (Figure 5F). C57 mice with bilateral 4T1 tumors were randomly divided into four groups. We found that HMP1G NPs treatment not only effectively inhibited the growth of primary tumors but also demonstrated the most significant inhibitory effect on the growth of distant tumors (Figure 5H,I). In contrast, the inhibitory effects of the 1‐MT and HMPG NPs treatments on both primary and distant tumors were limited, highlighting a lower ICB response rate when 1‐MT was used alone in vivo. Notably, the HMP1G NPs group showed the most effective inhibition of 4T1 tumor growth, with a median survival time of 38 days, which was significantly longer than that of the other groups. These results indicated that HMP1G NPs can achieve efficient ICB anticancer efficacy, eliciting more effective distal effects to inhibit tumor metastasis.

### 2.5 HMP1G NPs Ameliorate Immunosuppression in 4T1‐Tumor‐Bearing Mice

**Figure 6:**
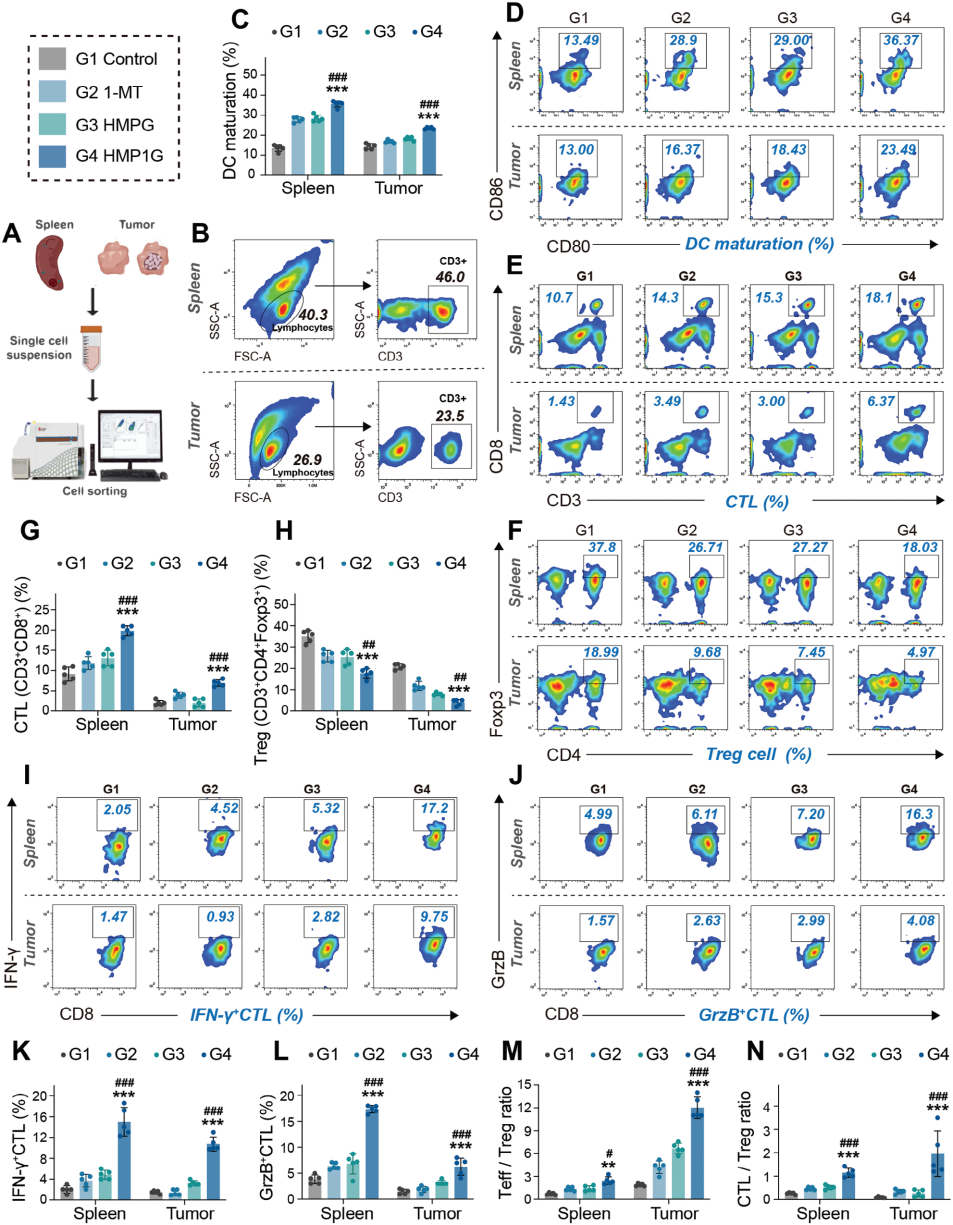
HMP1G NPs reprogrammed the immunosuppression of 4T1 tumor‐bearing mice. A) Schematic representation of the experimental setup for immune FCM analysis of spleens and distant tumors in the bilateral mouse model. B) Illustration of gating strategies for single‐cell FCM of spleens and tumors. D) Pseudocolor FCM plots and C) corresponding quantitative analysis of mature dendritic cells (CD11c^+^CD80^+^CD86^+^) in the spleens and tumors of different groups of mice. E) Pseudocolor FCM plots and G) corresponding quantitative analysis of CTLs (CD3^+^CD8^+^) in the spleens and tumors of different groups of mice. F) Pseudocolor FCM plots and H) corresponding quantitative analysis of Treg cells (CD3^+^CD4^+^Foxp3^+^) in the spleens and tumors of different groups of mice. I) Pseudocolor FCM plots and K) corresponding quantitative analysis of IFN‐γ^+^ CTL cells (CD3^+^CD8^+^IFN‐γ^+^) in the spleens and tumors of different groups of mice. J) Pseudocolor FCM plots s and L) corresponding quantitative analysis of GrzB^+^ CTL cells (CD3^+^CD8^+^GrzB^+^) in the spleens and tumors of different groups of mice. H) Teff/Treg ratio and CTL/Treg ratio in the spleens and tumors of mice. The source files were analyzed using FlowJo software. All data are presented as mean ± standard deviation (n = 6), where ^*^ is the comparison between this group and the control group, # is the comparison between this group and the 1‐MT group, N.S. represents the comparison between this group and the control group is meaningless, ^*^ *p* ≤ 0.05, ^**^ *p* ≤ 0.01, ^**^ *p* ≤ 0.001; # *P* ≤ 0.05, ## *p* ≤ 0.01, ### *p* ≤ 0.001.

The acidic environment and metabolic disturbances in the TME are the driving forces behind tumor immune suppression. We further evaluated the immune status of mice with bilateral tumor models using FCM after four days of different treatments. We collected mouse spleens and distant tumors to carefully assess the activation of immune cells in the spleen and the infiltration efficacy into the tumor bed by HMP1G NPs to investigate whether they could effectively reverse the tumor immune suppression status in 4T1‐tumor‐bearing mice (**Figure** **6**A,B). Immune evaluation groups were consistent with the treatment groups. The differentiation of T cells into functional CTLs and memory cells requires antigen recognition delivered by DCs, synergistic signaling, and cytokine stimulation.^[^ ^41^, ^42^ ^]^ Unexpectedly, we found that HMP1G NPs treatment led to effective maturation of DCs, with an abundance of 36.37% in the spleen, which was significantly higher than 28.9% abundance in the 1‐MT group, 29% in the HMPG NPs treatment group, and 13.49% in the untreated mice (Figure 6C,D). Simultaneously, mice treated with HMP1G NPs showed the most effective suppression of Treg cell populations (CD3^+^CD4^+^Foxp3^+^) both in the spleen and within the tumor, whereas 1‐MT and HMPG NPs treatments only slightly inhibited Treg cell populations within the tumor (Figure 6F,H).

Furthermore, while the impact of HMP1G NPs treatment on the frequency of CD3^+^CD8^+^ CTLs in the spleen and tumor was minor, slightly higher than the frequencies in the other treatment groups (3–4%) (Figure 6E,G), it may have led to a significant increase in specific types of CTLs infiltrating the tumor. As seen in Figure 6I–L, compared to untreated tumors, HMP1G NPs treatment resulted in an ≈8.50‐fold increase in the number of tumor‐infiltrating IFNγ^+^ CTLs (CD3^+^CD8^+^IFNγ^+^) and a threefold increase in the number of tumor‐infiltrating GrzB^+^ CTL (CD3^+^CD8^+^GrzB^+^), which are essential for CTL‐mediated potent anticancer cytotoxicity and immune activation.^[^ ^42^ ^]^ Based on the above analysis, it can be concluded that the abundance of effector T cells (CD3^+^CD4^+^) and cytotoxic T cells (CD3^+^CD8^+^) were significantly increased in mouse tumors treated with HMP1G NPs. Along with the reduction in the number of Treg cells in the tumor, this strategy resulted in a significant increase in the ratio of Teff cells to Treg cells, as well as the ratio of CTLs to Treg cells, which is an important marker of the activating antitumor adaptive immunity^[^ ^34^, ^42^ ^]^ (Figure 6M,N). Overall, the application of HMP1G NPs in 4T1‐tumor‐bearing mice triggered a strong antitumor immune response, as evidenced by DC maturation, infiltration of CTL, and a decreased Treg cell population within the tumor. These results confirmed that HMP1G NPs therapy can reverse the immunosuppressive TME to immunostimulatory effects, which may be attributed to the efficient anticancer effect mediated by CDT mediated by HMP1G NPs and excellent ICB sensitivity.

### 2.6 HMP1G NPs Ameliorated Immune Infiltration and Enhanced ICB Sensitivity Through the Ahr/STAT3/IL Axis in the Murine Tumor Model

**Figure 7:**
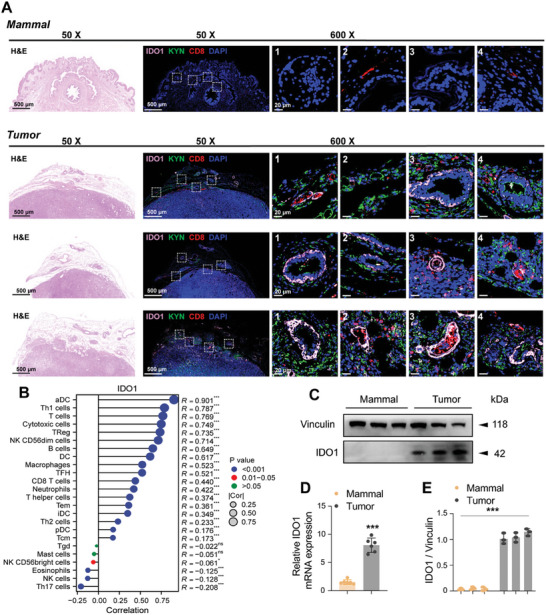
The upregulation of IDO1 protein expression in the BRCA mice Model is associated with immune infiltration. A) Representative brightfield H&E images and CLSM mIF images of normal mouse mammary glands and three typical cases of mouse BRCA. Alexa Fluor 648 was used to label IDO1, Alexa Fluor 488 for KYN, Alexa Fluor 555 for CD8, and DAPI for nuclear staining. (Scale bar: 500, 20 µm) B) Bubble plot showing the correlation of IDO1 protein with immune cells in the GEO dataset. C) and E) Immunoblotting and quantification of IDO1 protein levels, as well as D) IDO1 mRNA levels, in normal mouse mammary gland tissue and mouse mammary carcinoma tissue. All data are presented as mean ± standard deviation (n = 3, 6). ^*^ denotes comparisons between the gray and yellow groups, with significance levels of ^*^ *p* ≤ 0.05, ^**^ *p* ≤ 0.01, ^***^ *p* ≤ 0.001.

To explore the reasons for HMP1G NPs‐mediated unique ability to remodel the tumor immunosuppressive microenvironment thereby inhibiting the growth of primary and distant metastatic tumors in the BRCA mouse model, we first analyzed the effects of IDO1 and its metabolites on the mammary microenvironment in both normal and tumor tissues of the mice. Pathological identification and multiple immunofluorescence (mIF) determination of IDO1, KYN, and CD8^+^ T cells were performed after we collected normal mouse mammary glands and tumor‐bearing mouse mammary glands. As shown in **Figure** **7**A, normal mammary gland tissues exhibited regular cell morphology, a uniform distribution of ducts, no expression of the metabolic inhibitory IDO1 and KYN, and only a small amount of CD8^+^ T immune cell infiltration. In contrast, hematoxylin and eosin (H&E) staining revealed significant cellular atypia, nuclear pleomorphism, and mitotic figures in the three typical BRCA cases, whereas mIF demonstrates high levels of IDO1 and KYN expression, accompanied by partial CD8^+^ T cell infiltration. Analysis of the Gene Expression Omnibus database revealed a significant correlation between IDO1 protein levels and the number of immune cells (Figure 7B). This suggests that, for immunoinflammatory BRCA, the coexistence of immune cells and immunosuppressive factors may mediate antitumor immune inefficiency. Meanwhile, we aimed to delineate the causes of TME immunosuppression by collecting normal mouse mammary glands and tumor‐bearing mouse BRCA tissues for qualitative and quantitative analysis of IDO1. Quantitative real‐time PCR (qRT‐PCR) and WB results showed that compared to normal breast tissue, IDO1 protein, and mRNA levels in breast tumor tissue were significantly upregulated (Figure 7C–E). In conclusion, we found that IDO1^high^ BRCA patients had worse survival rates. IDO1 is closely related to immune cells, among which DC and T immune cells are the most closely related. The high expression levels of IDO1 in mouse breast cancer tumor tissues in vivo cannot be ignored. These data suggest that IDO1 derived from tumor cells may be a source of KYN/TRP metabolic disorders and may mediate TME immunosuppression.

**Figure 8:**
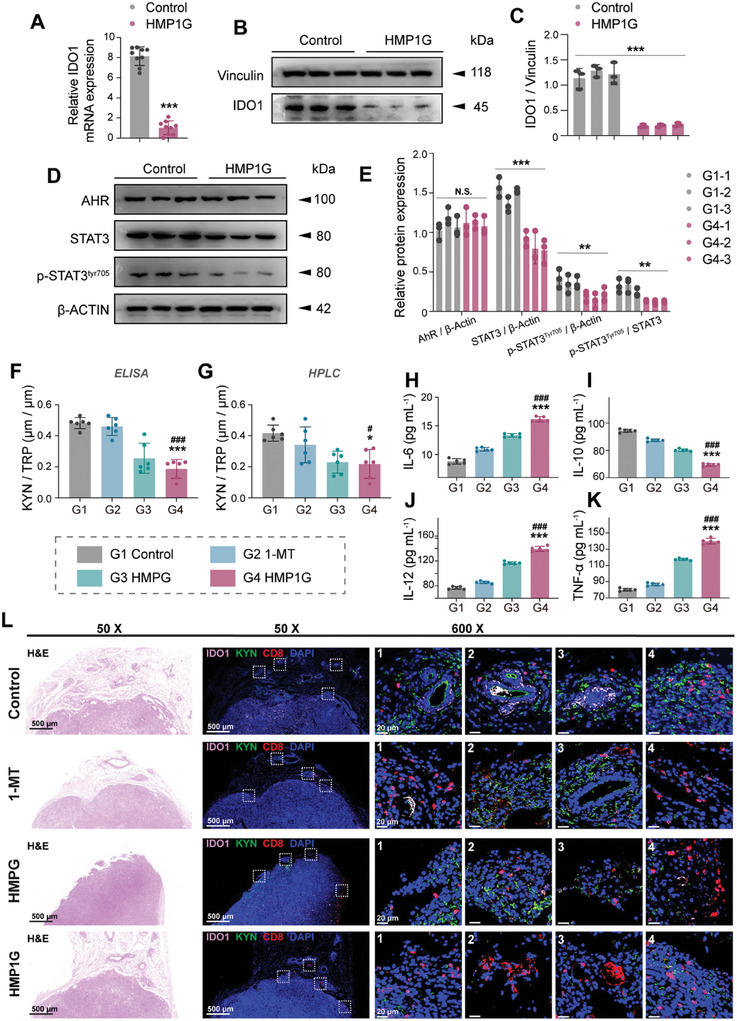
NPs ameliorate KYN/TRP metabolism and immune suppression by downregulating IDO1 through the AHR/STAT3/IL axis in vivo. Qualitative and quantitative analysis of IDO1 mRNA and protein levels in tumor tissues from different treated mice by using qRT PCR A) and Western blotting B) C) (n = 3). D) Corresponding quantitative analysis of AHR, STAT3, and p‐STAT3^Tyr705^ protein levels in tumor tissues from different treated mice by using immunoblot, and (E) respective quantification. F) ELISA and G) HPLC‐MS/MS were employed to determine the levels of KYN and TRP in the blood of mice from different treatment groups, and the corresponding KYN/TRP ratio was calculated (n = 6 biological replicates). Analysis of the secretion levels of H) IL‐6, I) IL‐10, J) IL‐12, and K) TNF‐α inflammatory factors in 4T1 tumor tissues of mice from different treatment groups using ELISA (n = 6). L) Representative immunofluorescence images of mouse tumor tissues stained with anti‐IDO1 (labeled with Alexa Fluor 648), anti‐KYN (labeled with Alexa Fluor 488), and anti‐CD8 (labeled with Alexa Fluor 555) antibodies are shown (scale bar: 500, 20 µm). All data are presented as mean ± standard deviation (n = 3, 6). ^*^ denotes comparison between the group and the control group, # denotes comparison between the column group and the 1‐MT group, N.S. indicates a non‐significant difference between the group and the control group, ^*^ *p* ≤ 0.05, ^**^ *p* ≤ 0.01, ^***^ *p* ≤ 0.001; # *p* ≤ 0.05, ## *p* ≤ 0.01, ### *p* ≤ 0.001.

The next question was whether HMP1G NPs improve KYN/TRP metabolism and immunosuppression in vivo, along with the specific mechanisms. To this end, we collected tumor tissue from the murine models at the end of the treatment cycle. As expected, the HMP1G NPs treatment group exhibited decreased protein and mRNA levels of IDO1 (**Figure** **8**A–C). While AhR protein levels showed no significant difference, total STAT3 and p‐STAT3^Tyr705^ levels were significantly downregulated in the tumor tissue of mice treated with HMP1G NPs (Figure 8D,E). Notably, therapeutic application of HMP1G NPs also significantly increased the secretion of IL‐6, IL‐12, and TNF‐α, within the tumor, indicating activation of antitumor immunity (Figure 8H,J,K), and decreased the expression level of IL‐10 (Figure 8I), a factor known to promote the differentiation of naïve CD4^+^ T cells into Treg cells.^[^ ^43^, ^44^ ^]^ This confirmed that HMP1G NPs promoted the high secretion of pro‐inflammatory cytokines and decreased the expression levels of anti‐inflammatory factors. This corroborates our cellular findings that HMP1G downregulates the expression of IDO1 via the AhR/STAT3/IL axis. IDO1 catalytic activity is directly proportional to the KYN/TRP ratio within the TME. We further investigated the levels of TRP and KYN in the 4T1 mouse model, collecting drops of blood and using ELISA and HPLC to evaluate the metabolic reversal efficacy of the NPs when applied in vivo. Our findings revealed a significant reduction in the KYN/TRP ratio in the tumors of mice treated with HMP1G NPs compared to those treated with 1‐MT alone (Figure 8F,G). These results indicated the effectiveness of HMP1G NPs at inhibiting IDO1 catalytic activity and decreasing the immunosuppressive KYN content within the blood.

**Figure 9:**
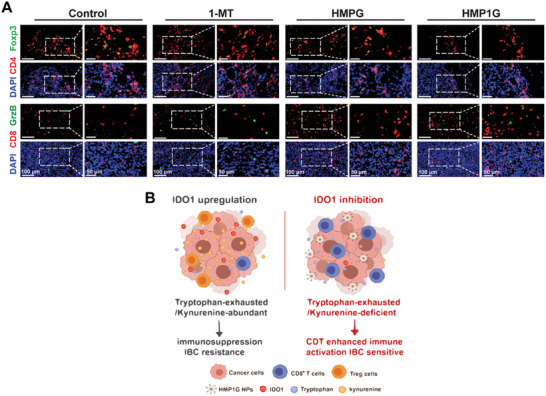
NPs ameliorate the T‐cell infiltration status in BRCA. A) Representative CLSM images of GrzB^+^CD8^+^ T cells (GrzB labeled with Alexa Fluor 488, CD8 labeled with Alexa Fluor 555) and FOXP3^+^CD4^+^ T cells (Foxp3 labeled with Alexa Fluor 488, CD4 labeled with Alexa Fluor 555) measured by IF staining in tumors. The magnified images of cells are shown within the white small boxes. (Scale bar: 10, 50 µm). B) Schematic illustration of the role of NPs in BRCA immune suppression and ICB resistance.

To explore the metabolic and immune statuses within the TME, we used mIF to detect the expression of IDO1, KYN, and CD8 in the tumor tissues of each group. As shown in Figure 8L, although the 1‐MT group effectively decreased IDO1 levels, the coexistence of KYN and CD8 still led to immunosuppression. In contrast, the HMP1G NPs group showed abundant CD8^+^ T cell infiltration in the field of view with minimal levels of IDO1 and KYN, suggesting a significant improvement in the metabolic and immunosuppressive states within the tumor microenvironment following HMP1G NPs treatment. To further assess the immune infiltration status within the TME, we conducted mIF staining analysis using anti‐CD4, anti‐Foxp3, anti‐CD8, and anti‐GrzB antibodies. The HMP1G NPs group exhibited a significant CD8^+^ T cell infiltration in the tumor bed, along with an increase in GrzB levels (**Figure** **9**A), and a decrease in the number of CD4^+^ T cells and the level of Foxp3. This improvement in immune activation far surpassed that in the free single‐agent 1‐MT and HMPG groups. Based on these results, we conclude that the high expression levels of IDO1 in 4T1 tumors are negatively correlated with antitumor immune infiltration, further supporting the view that the application of NPs can enhance the sensitivity of ICB therapy. In summary, our data confirmed that HMP1G NPs downregulate IDO1 expression via the AhR/STAT3/IL axis, suppress KYN/TRP metabolism, promote the secretion of antitumor inflammatory factors, and significantly enhance CTL cell infiltration to boost the ICB response sensitivity (Figure 9B), thus providing reliable experimental evidence for the clinical application of HMP1G NPs.

## 3 Discussion

In this study, we prepared a TME‐responsive HMP1G nanoplatform to enhance immunogenicity and ameliorate KYN metabolic dysregulation (two immunosuppressive factors within the TME of various solid tumors) to counteract the negative impact on the efficacy of ICB. The HMP1G NPs exhibited excellent TME‐dependent dissociation properties and 1‐MT and GSNO‐release behavior, which have been demonstrated to be effective at reversing the dysregulation of KYN metabolism and immunosuppression.

KYN is a host‐derived metabolite that contributes to tumor progression, immune evasion, and Treg cell development.^[^ ^11^, ^45^ ^]^ The conversion of TRP to KYN is regulated by IDO1 and the KYN/TRP ratio serves as a prognostic indicator for the response to ICB therapy.^[^ ^46^ ^]^ Generally, more than 95% of dietary tryptophan is metabolized via the KYNurenine pathway. Three distinct dioxygenases, tryptophan 2,3‐dioxygenase (TDO), IDO1, and IDO2, are responsible for the breakdown of TRP into KYN.^[^ ^47^ ^]^ TDO is primarily expressed in the liver, skeletal muscles, placenta, and brain. IDO2 is a paralog of IDO1 and is constitutively expressed in the liver, renal tubules, sperm, and DCs, whereas IDO1 exhibits a more ubiquitous expression pattern.^[^ ^48^ ^]^ Moreover, IDO1 is strongly associated with pathological conditions, especially in individuals with epithelial‐derived malignant tumors. Targeting IDO1 serves as a potential adjuvant to current immunotherapies;^[^ ^49^ ^]^ however, IDO1 enzyme inhibitors, such as epacadostat, have failed to demonstrate therapeutic benefits when used in combination with ICB in phase III trials for melanoma.^[^ ^50^ ^]^ The “off‐target” effects of IDO1 inhibitors are considered the primary reason for their clinical failures, with potential off‐target effects including compensatory activation of TRP analogs in somatic cells to activate AhR signaling and mechanistic activation of the rapamycin kinase (mTOR) signal.^[^ ^51^, ^52^ ^]^ Therefore, these compounds may trigger a range of AhR‐mediated effects in addition to inhibiting IDO1 enzyme activity.

The nano‐system we developed holds significant potential for enhancing both intra‐ and extra‐ antitumor immunity. The uptake efficiency and bioavailability of free 1‐MT are limited by its poor solubility. In contrast, HMP1G NPs with higher drug‐loading capacities effectively overcome the poor solubility of 1‐MT, enhancing drug bioavailability. At the cellular level, 4T1 cells efficiently internalized HMP1G NPs to the additional ·OH generated intracellularly in response to GSH and H_2_O_2_, initiating efficient CDT anticancer effects and releasing 1‐MT and GSNO. Simultaneously, Mn^2+^ triggered the decomposition of GSNO to release NO, which reacted with ROS to generate the more potent radical, ONOO^−^. The released NO, in collaboration with the small molecule, 1‐MT, collectively inhibited IDO1 expression. When co‐cultured with immune cells, the combined action of 1‐MT and NO significantly inhibited IDO1‐mediated tryptophan metabolism to suppress KYN production, activate DCs, and enhance T‐cell proliferation, thereby reversing tumor immune suppression. In the animal model, HMP1G NPs improved the metabolism of the immunosuppressive TME, reprogrammed it into an immunogenic phenotype, enhanced DC antigen presentation efficiency, activated CTL function, suppressed negative regulation by Treg cells, and increased the sensitivity to anticancer immune responses. From a mechanistic perspective, we revealed that BRCA tumors characterized by the overexpression of IDO1 have excessive KYN production through TRP degradation in an acidic TME, exacerbating metabolic dysregulation and immune suppression. The successful construction of the multifunctional HMP1G nanoplatform significantly decreased IDO1 protein levels without the off‐target effect of AhR activation, as observed with traditional IDO1 inhibitors. In conclusion, HMP1G NPs downregulated tumor‐derived IDO1 the AhR/STAT3/IL axis, improving KYN/TRP metabolism dysregulation, effectively overcoming metabolic immune suppression within the TME, reversing immunological tolerance in cold tumors, and sensitizing tumor cells to ICB therapy.

HMP1G NPs hold significant promise for enhancing ICB therapeutic strategies for poorly immunogenic tumors (such as BRCA). Given that inefficient aerobic glycolysis and KYN accumulation within tumors are common metabolic features of solid tumors, this study underscores a general and effective strategy for enhancing treatments for a broad range of tumors, particularly those with higher inherent immunogenicity. As the TME response and KYN elimination are highly effective at promoting the restoration of tumor immune surveillance, the nano‐platform strategy constructed in this study also holds promise for enhancing cancer immunotherapies and many other cancer treatments involving antitumor immunity.

## 4 Experimental Section

### Materials

TEOS, PAH, (molecular weight: 15000), PAA (molecular weight: 2000), and 1‐(3‐dimethylaminopropyl)‐3‐ethylcarbodiimide hydrochloride (EDC) were purchased from Aladdin Ltd. (Shanghai, China). Amino‐terminated poly (ethylene glycol) (PEG5k‐NH_2_, MW: 5000) was provided by RuiXi Biotechnology Ltd. (Xi'an, China). The sodium carbonate solution used in the study was obtained from Solarbio life science (Beijing, China). Kynurenine was acquired from Aladdin Ltd (catalog no. S138168; Shanghai, China). IFN‐gamma was from Abclonal Technology (catalog no. RP01070; Wuhan, China). Indoximod was acquired from MedChemExpress (catalog no. HY‐16724; USA).

### Cell Lines and Animals

The 4T1 cells, HC11 cells, HUVECs, DCs, and T cells, and their respective complete culture media were sourced from Prunus Company (Wuhan, China). All cells were cultured at 37 °C in a humidified atmosphere containing 5% CO_2_. Female C57BL/6 mice (6–8 weeks old) were obtained from the Animal Center of Chongqing Medical University. The mice were kept under specific pathogen‐free conditions with a 12‐h light/dark cycle, a temperature of 20 ± 3 °C, and a relative humidity of 40–70%. All animal experiments were conducted following the policies of the National Health Commission and approved by the Institutional Animal Care and Use Committee of Chongqing Medical University (approval no. 2022‐K332).

### Synthesis of HMP NPs and HMP1G NPs

Solid silica nanoparticles (sSiO_2_) were synthesized following the reported method.^[^ ^29^ ^]^ In Brief, a mixture of ethanol (14 mL), deionized water (2 mL), and ammonia (500 µL) was vigorously stirred in a 45 °C water bath for 20 min. Subsequently, 0.5 mL TEOS was added dropwise to this solution while maintaining continuous stirring at 150 rpm under a temperature of 50 °C for 1.5 h to obtain sSiO_2_. The as‐prepared sSiO2 was purified and dispersed in DI water.

Then, 10 mL of KMnO_4_ solution (30 mg mL⁻¹) was dropwise added into the suspension of sSiO_2_ (15 mg mL⁻¹) under ultrasonication. After 6 h of stirring, the precipitate was collected through centrifugation at 14800 rpm. The resulting mesoporous MnO_2_‐coated sSiO_2_ was dissolved in 2 M Na_2_CO_3_ and reacted at 60 °C for 12 h to yield hollow manganese dioxide (HMnO_2_).

The obtained HMnO_2_ were subjected to multiple cycles of centrifugation and washing with water, followed by freeze‐drying and subsequent redissolved post‐quantification. To enhance biostability, 5 mL of HMnO_2_ (2 mg mL⁻¹) was then added to 10 mL of PAH (5 mg mL⁻¹) under ultrasonication. After 2 h of stirring (800 rpm), the solution was centrifuged and washed with water at 148oo rpm for 10 min. The resultant HMnO_2_/PAH solution was gradually introduced into 10 mL of PAA solutions (5 mg mL⁻¹) under ultrasonication and with stirring (800 rpm) for 2 h, finally washing and collecting for further use. Next, PEG was modified onto the surface of HMnO2/PAH/PAA using the carbodiimide method. Following the addition of PEG5K‐NH_2_ (50 mg) under ultrasonication for 30 min. Subsequently, EDC (20 mg) was added, and the solution was stirred for 12 h, after which PEG‐ HMnO_2_ (HMP) was harvested via centrifugation (14,800 rpm for 10 min) and washed three times with water. The synthesis of HMP1G nanoparticles involved the incorporation of 1‐MT and GSNO into HMP, wherein the HMP solution was mixed proportionally with 1‐MT and GSNO under magnetic stirring overnight, yielding HMP1G NPs for use in subsequent experiments.

### Characterization of NPs

The morphology and elemental composition of nanoparticles were observed using transmission electron microscopy (TEM, FEI Tecnai G2 F30). The particle size and ζ potential were measured using a laser diffraction particle size analyzer (Zetasizer Nano ZS90). The amount of incorporated inorganic PEG components was determined by TGA (TGA 5500). The UV–vis spectra were recorded using a UV–vis–NIR spectrophotometer (PE Lambda 950). For porosity analysis, HMnO₂ was thoroughly washed with deionized water to remove surface impurities and unreacted chemicals, and then the sample was dried to constant weight. The specific surface areas and pore size were measured by using Brunauer–Emmett–Teller (BET; Micromeritics ASAP 2460, USA). XPS (Thermo escalab 250Xi) was employed to analyze the oxidation states of the manganese ions. Confocal laser scanning microscopy (CLSM, Nikon) was used to observe the fluorescent slices. The degradation behavior of HMP NPs was evaluated in different buffer solutions (pH 7.4, 6.5, 6.5 + 1 GSH 5 mm) and determined using UV–vis spectroscopy at different time points (0.5, 1, 2, 4, 12, 24 h). The release characteristics of 1‐MT and GSNO from HMP1G NPs were analyzed at 37 °C in different buffer solutions (pH 7.4, 6.5, 6.5 + GSH 5 mm) and determined using UV–vis spectroscopy at different time points (0.5, 1, 2, 4, 12, 24 h). The loading of GSNO and 1‐MT to the HMP1G NPs was quantified by UV–vis spectroscopy based on the standard curves of GSNO at 335 nm and 1‐MTat 288 nm, respectively. The drug‐loading efficiency(DLE) was calculated using the following equations:

(3) $$\mathrm{EE}\left(\%\right)=\mathrm{Amount}\;\mathrm{of}\;\mathrm{loaded}\;\mathrm{drug}/\mathrm{Weight}\;\mathrm{of}\;\mathrm{drug}\;\mathrm{in}\;\mathrm{feed}\times 100\%$$

### Assessment of Chemodynamic Process of HMP1G NPs

A mixture of 10 µg mL^−1^ MB, 100 µm H_2_O_2_, 0.5 mm MnCl_2,_ and 25 mm NaHCO_3_/5% CO_2_ buffer solution was placed in an oscillator at 37 °C for 0.5 h. MB was used as an indicator of ·OH because it can adsorb oxidized ·OH. MB degradation was monitored using a UV–vis spectrophotometer (665 nm). To evaluate the effect of GSH on HMP1G NPs mediated CDT, MB degradation via the Fenton reaction induced by Mn^2+^ was evaluated in the absence of and the presence of GSH at different concentrations (1, 2, 5, 10 mm). Specifically, a mixture of GSH, 10 µg mL^−1^ MB, 100 µm H_2_O_2_, HMP1G NPs ([Mn] = 0.5 mm), and 25 mm NaHCO_3_/5% CO_2_ buffer was incubated at 37 °C for 0.5 h and then centrifuged to obtain the supernatant, which was analyzed using a UV–vis spectrophotometer.

### In Vitro Biocompatibility Evaluation of HMP NPs

To determine the biocompatibility of HMP NPs, hemolysis assays were conducted. Fresh red blood cells were extracted from healthy female C57BL/6 mice and resuspended in PBS to prepare a diluted red blood cell suspension. Subsequently, the diluted red cell suspension was mixed with different concentrations (5, 25, 50, 100, 200, and 400 µg mL^−1^) of HMP1G solution and incubated at 37 °C for 2 h. The absorbance of the supernatant was measured at 570 nm, and the hemolysis rate was calculated using the following formula:

(4) $$\begin{array}{ccc} \mathrm{hemolysis}\left(\%\right) & = & \left({\mathrm{OD}}_{\mathrm{sample}}-{\mathrm{OD}}_{\mathrm{negative}}\right)/\left({\mathrm{OD}}_{\mathrm{positive}}-{\mathrm{OD}}_{\mathrm{negative}}\right)\\ & & \times 100\% \end{array}$$

PBS was used as the negative control, whereas deionized water was used as the positive control. Eight‐week‐old healthy BALB/c mice were randomly divided into 5 groups (three mice per group). The non‐injection group served as a control, while the remaining 4 groups had serum and blood collected on days 1, 7, 14, and 21 after *i.v*. administation of HMP1G NPs. Biochemical and hematological analyses were performed using an automatic biochemical analyzer (Rayto, Chemray 240, China) and an automatic blood analyzer (Mindray, BC‐2800 VET, China). The main organs of the mice (lungs, heart, liver, spleen, and kidneys) were subjected to histopathological examination using H&E staining. Cell proliferation and toxicity assays were performed using a CCK‐8 assay kit (Beyotime, China). HUVECs and DCs were pre‐seeded in 96‐well plates overnight. Subsequently, different concentrations of HMP1G NPs (0, 3, 6, 12.5, 25, 50, 100, and 200 µg mL^−1^) were added to the cells and they were co‐incubated for 24 h. Cell viability was then determined by measuring the absorbance at 450 nm using a microplate reader (Thermo Multiskan FC, USA).

### In Vitro Cytotoxicity and Cellular Uptake Assay

In the cytotoxicity assay, the CCK‐8 and annexin/PI apoptosis assay kits (4A Biotech, China) were used to evaluate the in vitro antitumor effects of HMP1G NPs. Briefly, 4T1 cells were pre‐seeded in a 96‐well plate overnight and then incubated with different concentrations of HMP1G NPs (5, 10, 25, 50, and 100 µg mL^−1^). The OD values were recorded at 450 nm using a microplate reader to assess NPs cytotoxicity. For the apoptosis assay, treated 4T1 cells were stained for apoptosis, and the percentage of apoptotic cells was evaluated by FCM. In the cellular uptake assay, 4T1 cells were pre‐seeded in a 6‐well plate overnight and incubated with DiI‐labeled HMP NPs at a drug concentration of 50 µg mL^−1^. The cells were then collected at 0, 1, 4, 8, and 12 h, and the efficiency of the cellular uptake of NPs was observed using CLSM.

### Intracellular NO Release and ROS Generation

Intracellular NO levels were measured using a NO fluorescent probe (DAF‐FM DA; Beyotime). After incubating 4T1 cells under various treatment conditions for 24 h, the cells were washed with PBS, stained with a DAF‐FM DA solution (diluted 1:1000), and then subjected to FCM to determine the NO levels. Intracellular ROS levels were measured using a ROS probe (DCFH‐DA, Beyotime). After incubating 4T1 cells under various treatment conditions for 24 h, the cells were washed with PBS, stained with a DCFH‐DA solution (diluted 1:1000), and then subjected to both FCM and CLSM for qualitative and quantitative analyses of ROS levels.

### Immunofluorescence

Samples embedded in immunofluorescence paraffin were sectioned into 4 mm slices. Antigen retrieval was performed in a citrate solution using a microwave (95 °C, 30 min). For cell samples, cells (5 × 10^4^) were seeded on coverslips and fixed with fresh 4% paraformaldehyde in methanol for 15–20 min. The coverslips were then incubated in the dark at room temperature in PBS containing 2% bovine serum albumin for 1 h. All antibodies were purchased from ABCAM (Cambridge, UK). For multiplex immunofluorescence staining, the coverslips were incubated overnight at 4 °C in a mixture of two primary antibodies. The following primary antibodies were used: anti‐CD8 mouse antibody, anti‐IDO1 mouse antibody, anti‐KYN mouse antibody, anti‐FOXP3 rabbit antibody, anti‐GrzB rabbit antibody, and anti‐CD4 mouse antibody. After washing the coverslips with cold PBS, they were incubated in the dark at room temperature for 1 h with a mixture of two secondary antibodies raised against different species. The following secondary antibodies were used: Alexa Fluor 488‐labeled anti‐rabbit antibody, Alexa Fluor 555‐labeled anti‐rabbit antibody, Alexa Fluor 648‐labeled anti‐rabbit antibody, Alexa Fluor 488‐labeled anti‐mouse antibody, Alexa Fluor 555‐labeled anti‐mouse antibody, and Alexa Fluor 648‐labeled anti‐mouse antibody. Coverslips were counterstained with 4′,6‐diamidino‐2‐phenylindole (DAPI; Beyotime, China) for nuclear observation. Each sample was examined using a CLSM.

### qRT‐PCR Analysis

Total cellular RNA was extracted using TRIzol reagent (catalog no. 15596026; Invitrogen, USA) and reverse‐transcribed to cDNA using M‐MLV reverse transcriptase (catalog no. M1701, Promega, USA). qRT‐PCR was performed using SYBR Green (catalog no. HY‐K0501; MCE, USA) and run on a CFX96 real‐time system C1000 thermocycler (Bio‐Rad, USA) according to the manufacturer's instructions. The relative mRNA levels were calculated using the 2^ΔΔCt method with GAPDH as an internal control. The primer sequences are listed in Table S1 (Supporting Information).

### WB Analysis

The protein concentration was determined using the bicinchoninic acid method (Beyotime). All antibodies were purchased from ABCAM (USA). WB analysis was carried out using the following antibodies according to standard protocols: anti‐IDO1 mouse antibody, anti‐AHR rabbit antibody, anti‐STAT3 rabbit antibody, anti‐p‐STAT3^Tyr705^ rabbit antibody, anti‐β‐actin rabbit antibody, anti‐vinculin rabbit antibody, goat anti‐rabbit immunoglobulin G, and goat anti‐mouse immunoglobulin G. Protein band intensity analysis was performed using Image J 1.42q software (National Institutes of Health, Bethesda, MD, USA) to quantify the levels of each protein by determining the grayscale levels of each band and normalizing to β‐actin or vinculin.

### Detection and Analysis of Amino‐Acid‐Related Metabolites

Cells (2 × 10^7^ cells/well) were cultured under different conditions for 24 h. After removing the culture medium and replacing it with 10 mL of serum‐free DMEM, the supernatant was collected after 48 h and filtered through a 0.45 µm filter to remove any floating cells. For mouse plasma samples analysis, 100 ± 5 µL of mouse plasma was placed in a 2 mL centrifuge tube after thawing on ice. A TRP ELISA assay kit (ML077292; Enzyme‐Linked Biotechnology, China) and the KYN ELISA assay kit (YJ243862; Enzyme‐Linked Biotechnology, China) were used to analyze cells and mouse blood samples, following the manufacturer's instructions. The TRP and KYN levels were inferred from the respective known standards provided in each assay kit, and the numerical ratios (KYN/TRP) were calculated and plotted using GraphPad Prism software (version 8; Dan Diego, CA, USA). The amino acid contents of the cell samples and mouse blood samples were determined using HPLC‐MS/MS (Agilent HPLC 1260, USA). A 10 µL internal standard solution and 0.4 mL of acetonitrile‐methanol were added to the samples and mixed by vortexing. After centrifugation at 14000 × g, 400 µL of supernatant was collected and dried under nitrogen. The residue was dissolved in 100 µL of acetonitrile water, followed by centrifugation at 14000 × g. The supernatant was then injected into the HPLC‐MS/MS instrument for analysis. MultiQuant and Analyst software were used for quantitative data processing.

### Analysis of Immune Levels

The proliferation of mature DCs and T cells was assessed. 4T1 cells from each group were pretreated for 24 h, followed by co‐culturing with DC cells in a transwell system for an additional 24 h. Subsequently, the DCs were collected for surface staining with anti‐CD11C, ‐CD80, and ‐CD86 antibodies (all from Ebioscience, USA), and analyzed using FCM. The supernatant from the DC cell culture medium was collected and subjected to centrifugation, followed by ELISA to measure the secretion levels of IL‐6, IL‐12, IL‐10, and TNF‐α (Enzyme‐Linked immunosorbent assay, China). In the T cell proliferation experiment, the key difference was in the utilization of EDU to stain the T cells.

At the in vivo level, on the fourth day after treatment of the mice in each group, both the tumors and spleens were homogenized and enzymatically digested at 37 °C (collagenase I and hyaluronidase I, 1.5 mg mL^−1^) for 1 h in order to prepare corresponding single‐cell suspensions. Standard protocols were then employed for staining with appropriate FCM antibodies (all from Ebioscience, USA) to stain DCs (CD11c^+^CD80^+^CD86^+^) in the spleen and tumor, as well as Treg cells (CD3^+^CD4^+^foxp3^+^), CD8^+^ T cells (CD3^+^CD4^−^CD8^+^), IFN‐γ^+^ CTLs (CD3^+^CD4^−^CD8^+^IFN‐γ^+^), and GrzB+ CTLs (CD3^+^CD4^−^CD8^+^GrzB^+^) within the tumor. For cytokine detection, an ELISA kit was used to measure the secretion levels of IL‐6, IL‐12, IL‐10, and TNF‐α in the homogenized tumor tissues, following the manufacturer's instructions.

### Tumor Model and Treatment Experiments

To establish a unilateral subcutaneous tumor model, 1 × 10^6^ 4T1 cells were injected subcutaneously into the right flank of female C57 mice (6‐8 weeks old). Treatment commenced when the tumor size reached 130–150 mm^3^. The mice were then randomly divided into 4 groups, including the control group, 1‐MT group, HMPG group, and HMP1G group. Mice in groups 2–4 received NPs *i.v*. administration of NPs on days 0, 3, and 6 at equivalent doses of NPs (20 mg kg^−1^) and 1‐MT (6.6 mg kg^−1^), totaling 3 doses. Tumor volume was calculated using the formula length × width^2^ × 0.5. The Mice were euthanized when the tumors became necrotic or reached a volume of ≈1500 mm^3^, and these events were recorded on a survival curve. To establish a bilateral tumor model, distant tumors were formed 5 days after the primary tumor was established. When the size of the distant tumor reached ≈50–80 mm^3^, treatment was commenced with the same four‐group division as in the unilateral model. Subsequent treatment and follow‐up monitoring of the bilateral tumors followed the procedure outlined above. Upon completion of the follow‐up period, blood was collected from the mice for analysis using ELISA and HPLC‐MS/MS analyses of tryptophan and KYN metabolism levels. Tumors were also collected for FCM, H&E staining, and mIF analysis.

### Statistical Analysis

Quantitative data are presented as the mean ± standard deviation. When two or more groups were compared, statistical differences were calculated using an unpaired Student's *t*‐test or one‐way analysis of variance (ANOVA) via GraphPad Prism software (version 8). Survival curves were analyzed using the log‐rank test. All the experiments in the study were performed at least thrice and similar results were obtained. Statistical differences are denoted as follows: ns, not significant; When *p* < 0.05, the difference was considered significant.

## Conflict of Interest

The authors declare no conflict of interest.

## Author Contributions

M.N.W. and Y.H.L. contributed equally to this work. G.H.H and M.P. conceptualized supervised the project, and provided funding support; Y.S.L. and T.L. performed validation, and investigation and provided funding support. M.W., T.L.W., Z.B.C. and L.C. drafted and finalized the manuscript.

## Supporting information



Supporting Information

## Data Availability

The data that support the findings of this study are available from the corresponding author upon reasonable request.
