# Supplementary material for: Tumor Microenvironment‐Responsive Nanoparticles Enhance IDO1 Blockade Immunotherapy by Remodeling Metabolic Immunosuppression
Source: Adv Sci (Weinh). 2024 Dec 11;12(5):2405845. doi: 10.1002/advs.202405845 (PMC11791960; doi:10.1002/advs.202405845)
Supplement: Supplementary file 1 — Supporting Information [file ADVS-12-2405845-s001.docx]

**Supporting informations**


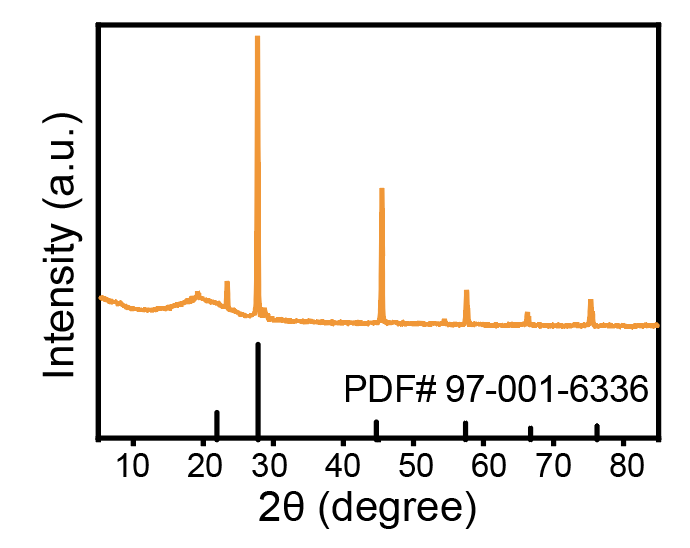


Figure S1. Representative XRD scan spectra of SiO_2_.


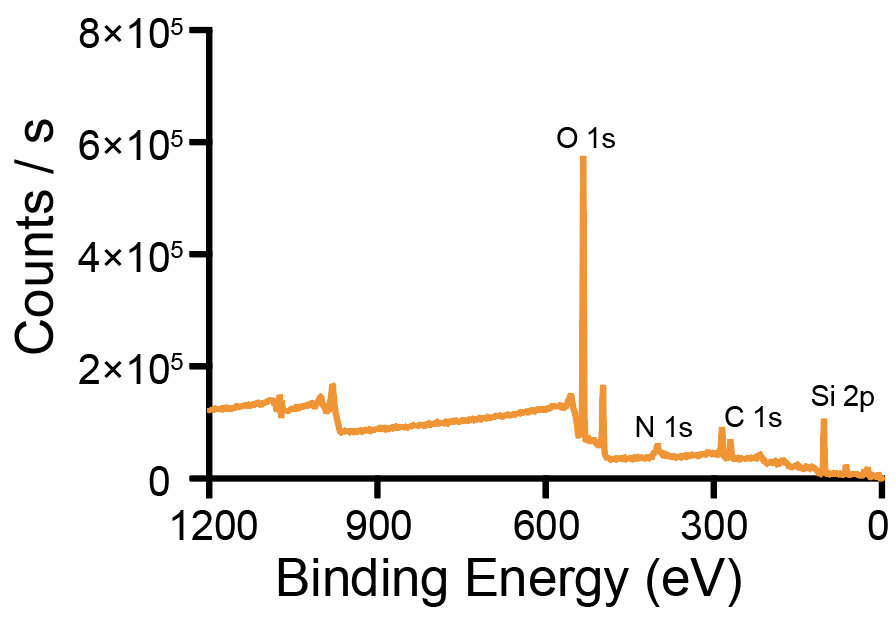


Figure S2. Representative XPS full scan spectra of SiO_2_.


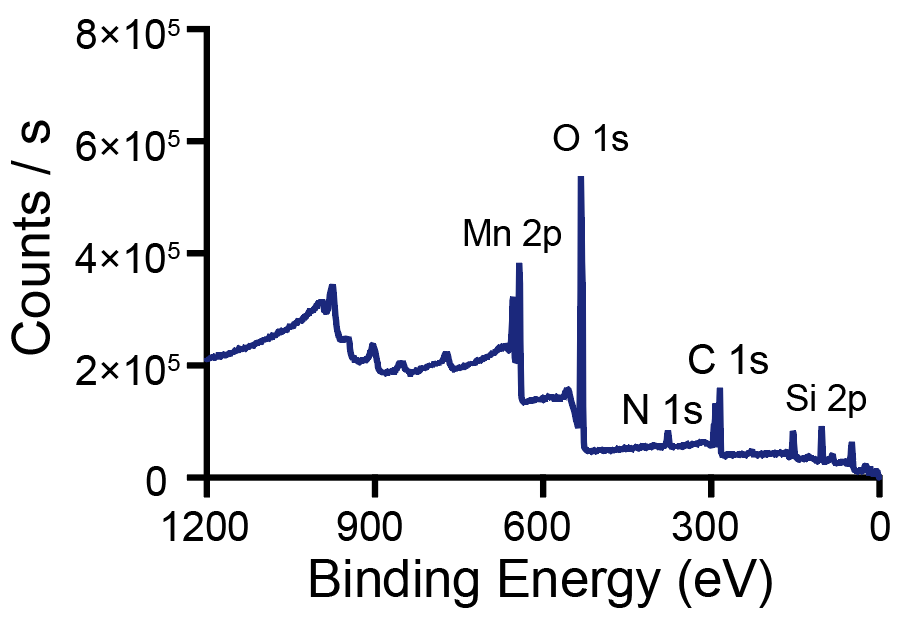


Figure S3. Representative XPS full scan spectra of SiO_2_-MnO_2._


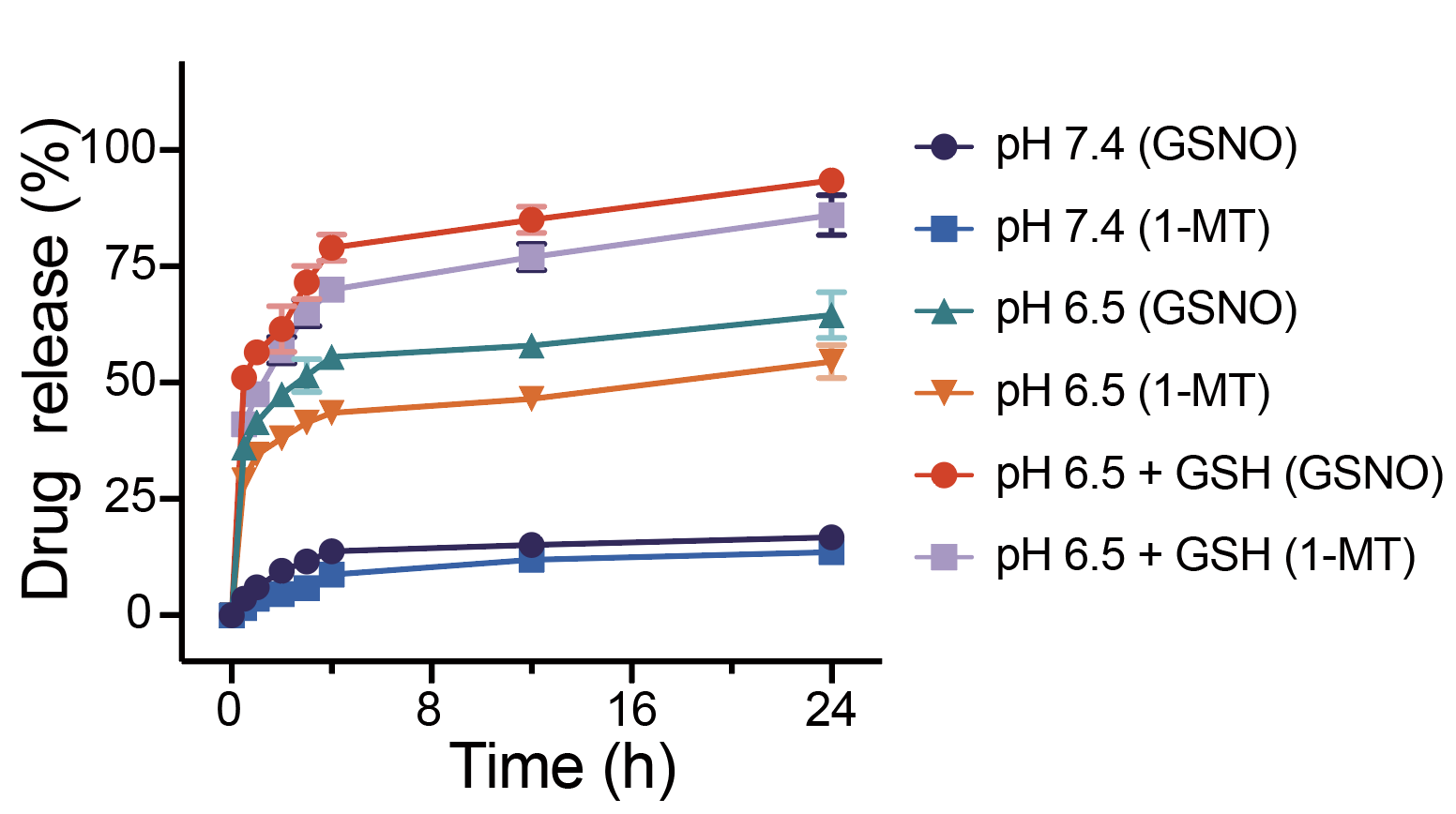


Figure S4 : Cumulative release kinetics of GSNO and 1-MT from HMP1G NPs in varied conditions. ( pH7.4, pH 6.5, pH 6.5 + [GSH 5 mM] ). Data are presented as means ± standard deviation (s.d.) (n = 3)


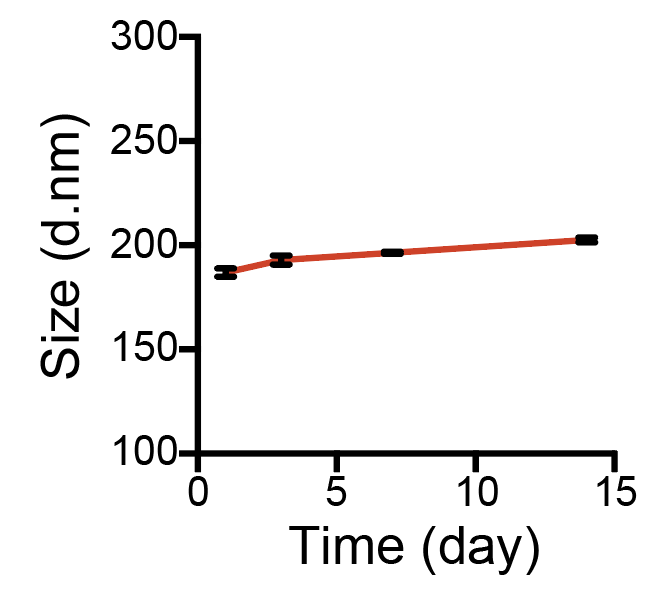


Figure S5. Particle size distribution of HMP1G NPs in culture medium over 14 days. Data are presented as means ± standard deviation (s.d.) (n = 3)


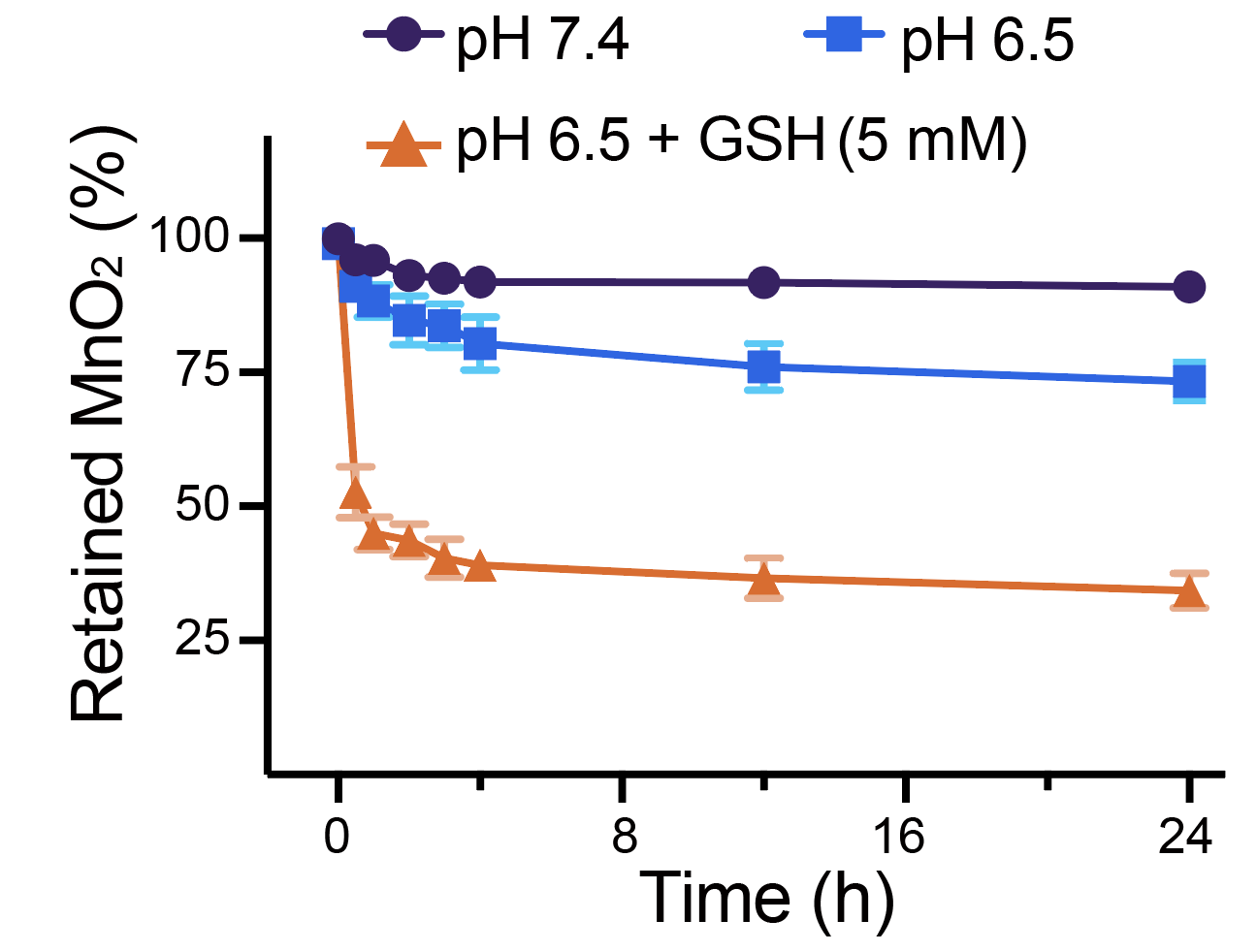


Figure S6. The degradation behavior of HMP NPs dispersed in PBS (pH 7.4), PBS (pH 6.5) and PBS (pH 6.5) containing GSH (5mM). Data are presented as means ± standard deviation (s.d.) (n = 3)


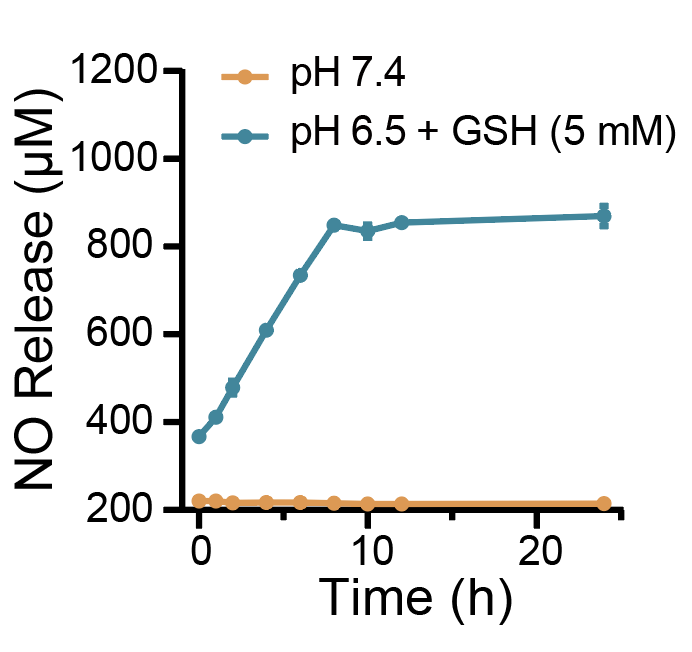


Figure S7. NO release profiles of HMP1G NPs in PBS containing or lacking GSH (5 mM) measured by Griess reaction.


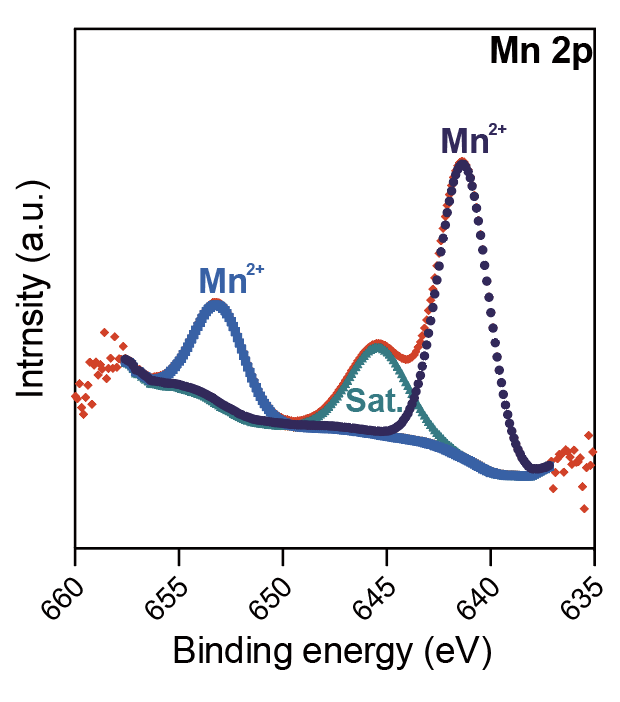


Figure S8. XPS spectra of *Mn 2p* region of HMP NPs under acidic condition. (PBS containing GSH [5mM] ).


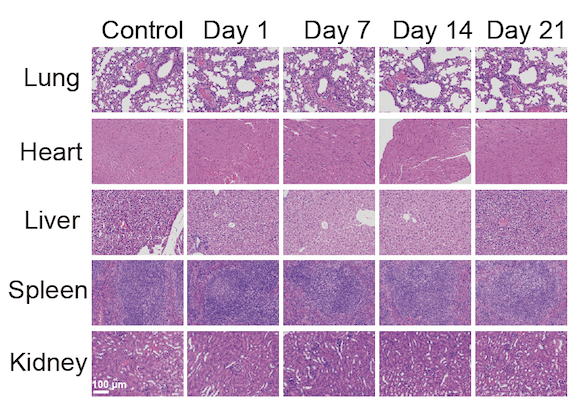


Figure S9. Representative H&E-stained tissue sections of major organs in different groups of mice (scale: 100 µm).


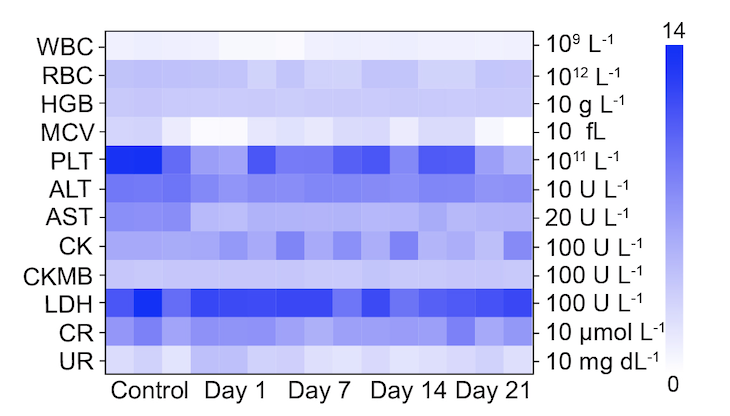


Figure S10. Hematological and serum biochemical analyses of blood samples from mice in various groups. Data are presented as means ± standard deviation (s.d.) (n = 3 independent animals).


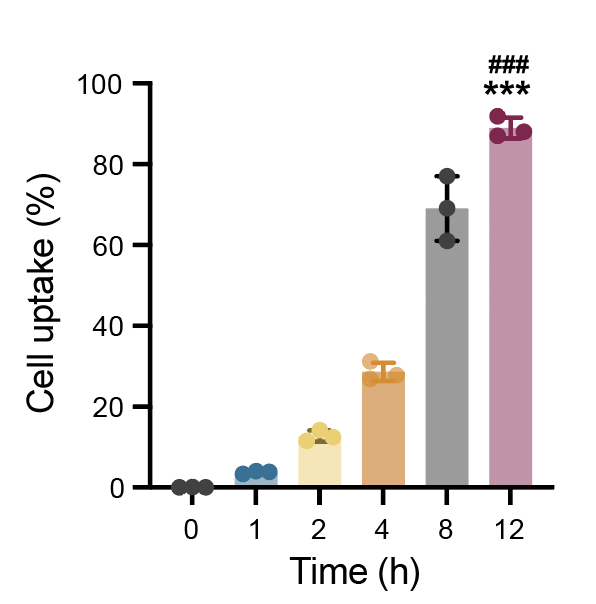


Figure S11. Flow cytometry analysis of NPs phagocytosis showing statistical values. Data are presented as mean ± standard deviation (n = 3). * denotes comparison between the group and control group, # denotes comparison between this group and the 1-MT group, N.S. indicates non-significant between this group and control group, * p ≤ 0.05, ** p ≤ 0.01, *** p ≤ 0.001; # p ≤ 0.05, ## p ≤ 0.01, ### p ≤ 0.001.


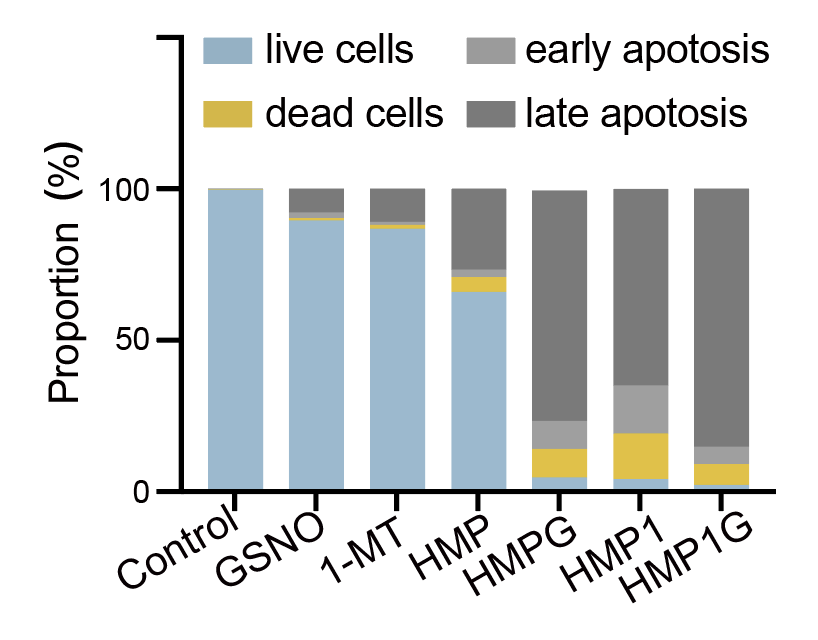


Figure S12. Statistical values of different stages of apoptosis in 4T1 cells 24 hours after treatment in different groups using flow cytometry.


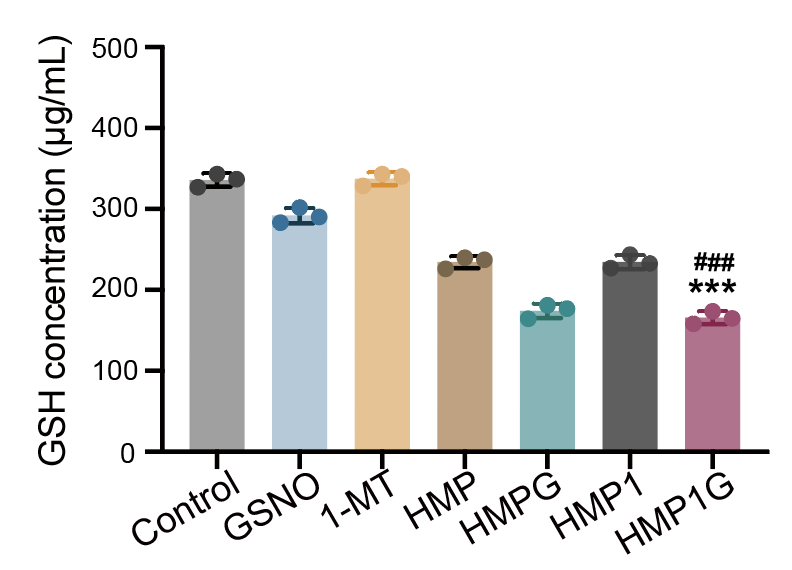


Figure S13. Quantification of intracellular GSH content in 4T1 cells 24 hours after treatment in different groups. Data are presented as mean ± standard deviation (n = 3). * denotes comparison between the group and control group, # denotes comparison between this group and the 1-MT group, N.S. indicates non-significant between this group and control group, * p ≤ 0.05, ** p ≤ 0.01, *** p ≤ 0.001; # p ≤ 0.05, ## p ≤ 0.01, ### p ≤ 0.001.


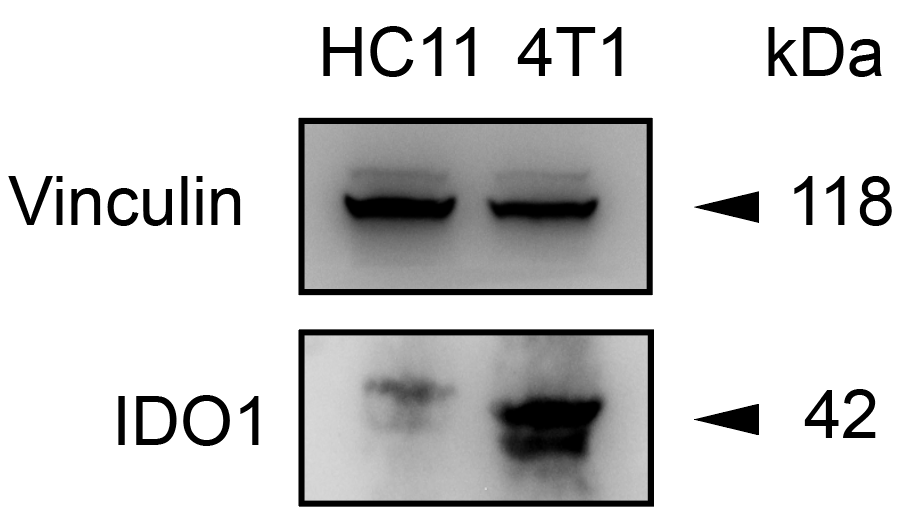


Figure S14. Immunoblotting of IDO1 protein level in normal mouse mammary epithelial cells (HC11) and mouse mammary carcinoma cells (4T1).


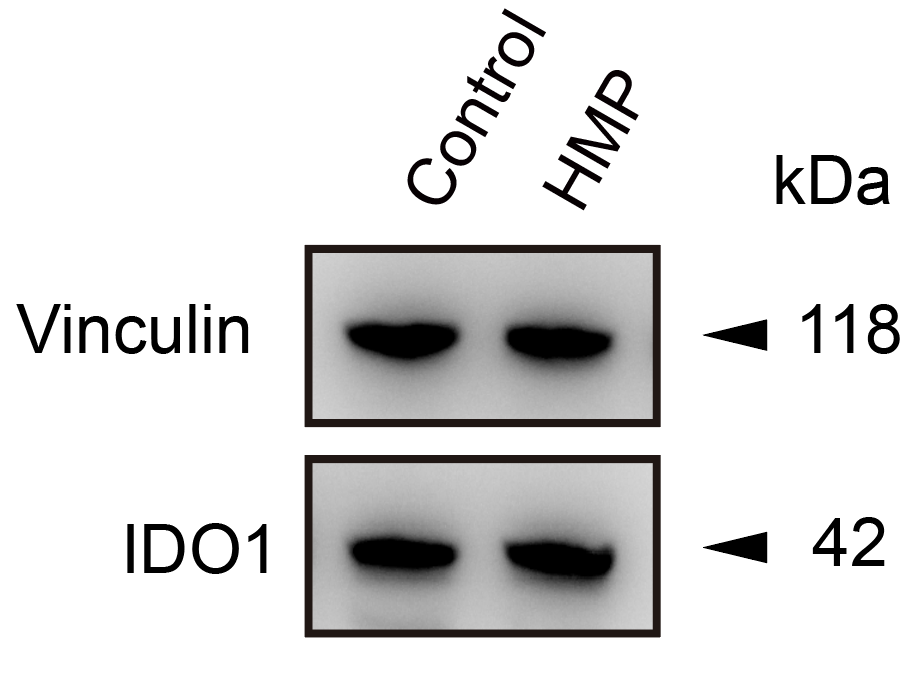


Figure S15. IDO1 protein expression in 4T1 cells treated with HMP NPs for 24 h, with PBS-treated cells as control.

| **Table S1. The lists of primers used for qRT-PCR.** | | |
| --- | --- | --- |
| **Primers Primers for qRT-PCR** | **Sequence (5'-3')** | |
| *ido1* (mouse) | Forward | GCAGACTGTGTCCTGGCAAACT |
|  | Reverse | AGAGACGAGGAAGAAGCCCTTG |
| *gapdh (mouse)* | Forward | CATCACTGCCACCCAGAAGACTG |
|  | Reverse | ATGCCAGTGAGCTTCCCGTTCAG |
